# A chromosome-scale genome assembly and functional studies implicate the viral RNA polymerase in transcription of most Microplitis demolitor bracovirus late genes

**DOI:** 10.1128/jvi.00506-26

**Published:** 2026-07-17

**Authors:** Kelly T. Eidson, Corinne M. Stouthamer, Tyler J. Simmonds, Sheina B. Sim, Scott M. Geib, Michael R. Strand, Gaelen R. Burke

**Affiliations:** 1 Department of Entomology, University of Georgia 1355, Athens, Georgia, USA; 2 Tropical Pest Genetics and Molecular Biology Research Unit, USDA-ARS Daniel K Inouye U.S. Pacific Basin Agricultural Research Center, USDA ARS 57524, Hilo, Hawaii, USA; Wageningen University & Research, Wageningen, Netherlands

**Keywords:** parasitoid, virus, bracovirus, endogenous virus element, genome, polymerase

## Abstract

Microgastroid parasitoids lay eggs in other insects and harbor bracoviruses (BVs), which evolved from a nudivirus. BV genomes are endogenized in the wasp germline and consist of nudivirus-like genes plus proviral segments with virulence genes. Nudivirus genes produce virions in the ovary calyx cells, which wasps use to transfer virulence genes to hosts. Here, we report a chromosome-scale genome assembly for *Microplitis demolitor,* which harbors Microplitis demolitor bracovirus (MdBV). The assembly identified the locations of all MdBV nudivirus-like genes and proviral segments. Like nudiviruses and related baculoviruses, BVs express nudivirus-like genes in a temporally ordered cascade. Early genes include subunits of an RNA polymerase (RNApol) that transcribes late genes encoding virion components. Promoter motifs in BV relatives did not predict MdBV early and late genes. In contrast, RNA interference experiments showed that knockdown of the MdBV RNApol downregulated most of the known nudivirus-like late genes. We identified 60 genes with no homology with known nudivirus genes, with features indicating that they were also inherited from the BV ancestor. We named these candidate, new nudivirus-like genes. Each was expressed as a late gene, 39 encoded virion components, and orthologs were identified in wasps that are closely related to *M. demolitor*. Finally, we identified a new 20 bp motif in most MdBV late genes. This motif was present in the nudivirus-like genes of another BV but was largely absent in nudivirus and baculovirus genes. Altogether, the results identified several new features of BVs that are important for virion formation.

**IMPORTANCE:**

Bracoviruses (BVs) provide a dramatic example of cooption and repurposing of a virus by eukaryotes for new functions. This study further characterizes the gene content and organization of the MdBV genome. Our results support the conclusion that the MdBV RNApol retains conserved functions by primarily transcribing viral late genes. Reported results also present a strategy for identifying BV genes that share no homology with known nudiviruses.

## INTRODUCTION

Viruses in the family Nudiviridae are subdivided into several genera that infect insects or other arthropods (1–3). Structural features of the family include a circular, double-stranded (ds) genome (96–232 kb, 87–154 intronless genes) and enveloped, rod-shaped nucleocapsids that assemble in the nuclei of infected host cells. All family members share 32 core genes, while other accessory genes are in some but not all genera, are genus-specific, or are known from only one or a few species. Arthropod-infecting viruses in the family Baculoviridae are the closest relatives of nudiviruses as evidenced by several traits, including 21 core genes that both families share: *dnapol* and *helicase* (replication of the viral genome); *lef-4, lef-8, lef-9,* and *p47* (subunits of an RNA polymerase [RNApol]); *lef-5* (initiation factor); *38K, p6.9, vlf-1,* and *vp39* (capsid components and genome packaging); *p74, pif-1* to *-6,* and *pif-8* (envelope components and infectivity factors); and *p33* and *ac81* (assembly factors) (1, 4).

Nudivirus-derived sequences have been identified in many arthropod genomes (5). Three different nudiviruses have also integrated into the germline of different parasitoid wasps and evolved into entities called domesticated endogenous viruses (DEVs) (6). Parasitoid wasps lay eggs in or on other arthropods that serve as hosts for offspring, while DEVs are characterized by the permanent integration of many nudivirus genes that are only expressed in calyx cells of pupal and adult stage females (7, 8). Calyx cells reside in a proximal domain of the ovaries called the calyx, while the expression of DEV gene products produces particles in the nuclei of calyx cells. Particles exit calyx cells and accumulate to high density in the lumen of the calyx, where they are referred to as calyx fluid. Females thereafter inject particles into hosts with eggs (6–8). DEV particles cannot replicate but have functions in hosts that enable wasp offspring to develop (9, 10). Reciprocally, vertical inheritance of DEV genome components assures that female offspring can produce particles in ovary calyx cells (6, 8).

Bracoviruses (BVs) are the largest of the three nudivirus-derived DEV lineages. BVs evolved ~100 million years ago from a nudivirus that integrated into a parasitoid in the family Braconidae (11, 12). Speciation thereafter produced a large monophyletic assemblage called microgastroid braconids (~50,000 species in six subfamilies) that primarily parasitize larval-stage moths (Lepidoptera) (13). BV particles assemble in calyx cell nuclei, contain circular dsDNAs, and share morphological similarities with nudivirus virions that include one or more nucleocapsids that are surrounded by a single envelope. Multiomic studies of microgastroids like *Cotesia congregata, Chelonus insularis,* and *Microplitis demolitor* indicate that BV genetic components consist of nudivirus genes and domains called proviral segments (14–17).

In the case of *M. demolitor*, the current Mdem2 genome assembly identifies 72 homologs of genes in known nudiviruses (15). Most are intronless and single copy, but some form families. Included are all core genes that nudiviruses share with baculoviruses except *dnapol* and *ac81,* while most of the other genes are named for the nudivirus homolog they are most similar to. Twenty of these genes reside in a domain called the nudivirus cluster, while 52 are elsewhere in the wasp genome. The nudivirus cluster also contains four genes (*K425_459, K425_438, K425_456,* and *K425_461*) that are absent in known nudiviruses but share features consistent with acquisition from the BV ancestor (14). We refer to the above inventory as the known nudivirus-like genes of M. demolitor bracovirus (MdBV). All are expressed in ovary calyx cells in a temporally ordered cascade, which is how baculovirus and nudivirus genes with functions in producing virions are expressed after infecting host cells (4, 9, 18). The MdBV RNApol subunits (*lef-4, lef-8, lef-9,* and *p47*) plus *lef-5, vlf-1a,* and *HzNVorf128-*like are classified as early genes because expression begins in early-stage wasp pupae, while the other 69 genes are classified as late because expression begins in mid-stage pupae (19, 20). Knockdown of MdBV RNApol subunit genes by RNA interference (RNAi) significantly reduces the expression of two late genes (*p74* and *vp39*) encoding virion components (21). Proteomic analysis of MdBV virions and functional experiments indicate that other late genes also encode structural proteins and/or are required for virion assembly (20–22).

Unlike nudiviruses, which amplify and package their genomes into capsids, sequences in the wasp genome called proviral segments are amplified and processed into the circular dsDNAs that are packaged into BV capsids (6, 9, 10). Proviral segments encode virulence genes with diverse origins but no recognizable nudivirus-like genes. The current *M. demolitor* genome assembly identifies 26 MdBV proviral segments, which contain a total of 96 virulence genes (15). Some of these genes are single copy, others form multimember families, and several contain small introns. MdBV proviral segments are organized into eight loci (also called replication units) that are amplified in calyx cell nuclei (14, 23, 24). Amplicons are then processed into the circularized dsDNAs that are packaged into capsids by nudivirus-like gene products in the integrase family (Vlf-1a, Vlf1b-1, Vlf1b-2, and Int-1) (20, 21, 25). After injection into hosts, MdBV virions infect hemocytes and other types of cells where virulence genes are expressed. The resulting products benefit wasp offspring by disabling host immune defenses and growth (26–30).

This study addresses three important knowledge gaps in our understanding of BVs generally and MdBV in particular. First, the Mdem2 assembly for the *M. demolitor* genome is scaffold level, with an N50 of 1.1 Mb (15). Most of the aforementioned MdBV genome components are present in the Mdem2 assembly, but the lack of information on the precise genomic locations of different nudivirus-like genes and proviral loci hinders the understanding of how BV and wasp components are organized at the chromosome scale. Second, the effects of MdBV RNApol knockdown on *vp39* and *p74* are consistent with this polymerase transcribing these late genes. In contrast, whether the MdBV RNApol regulates the expression of other genes is unknown. Third, while core genes are shared among all known nudiviruses or baculoviruses, other accessory genes range from being present in many but not all species to only being known from a single species or a small number of species within a given genus (1, 4, 31, 32). As noted above, *K425_459, K425_438, K425_456,* and *K425_461* are absent in known nudiviruses, but their structure and location in the nudivirus cluster of *M. demolitor* and select other microgastroids strongly suggest that they are accessory genes that were inherited from the BV ancestor (14–16). We thus reasoned that other accessory genes from the BV ancestor could also exist in the *M. demolitor* genome outside the nudivirus cluster. Here, we report a chromosome-level assembly for the *M. demolitor* genome. We then used this assembly in conjunction with other assays to show that the MdBV RNA polymerase (RNApol) regulates the expression of nearly all MdBV late genes that are required for virion formation. We also used the new genome assembly with a screening strategy that identified 59 genes in the *M. demolitor* genome outside the nudivirus cluster and 1 gene inside the nudivirus cluster that have no homology to known nudiviruses but share other features that strongly suggest that they derive from the BV ancestor. Many of the candidate, new nudivirus-like genes also encoded virion proteins and shared a novel promoter motif present in other nudivirus-like late genes that are regulated by the MdBV RNApol. Altogether, the integrative use of the multiomic data reported in this study significantly advances the understanding of BV genome organization, function, and evolution.

## RESULTS

### A chromosome-level genome assembly for *M. demolitor*

**Fig 1:**
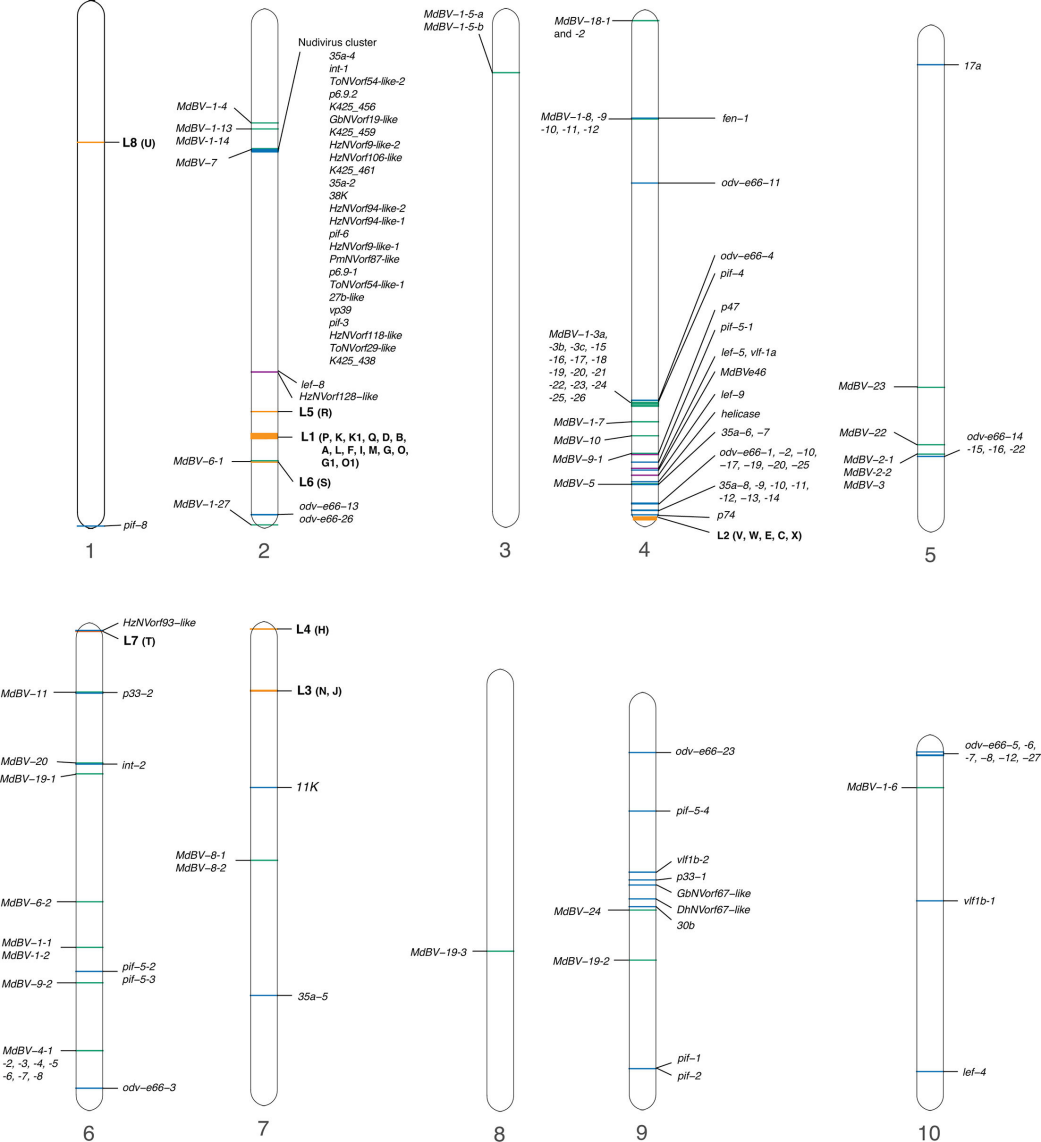
Schematic showing the locations of MdBV genome components on the 10 *M. demolitor* chromosomes. Known early (purple bars) and late (blue bars) nudivirus-like genes are identified to the right of chromosomes. The nudivirus cluster containing only late genes is also identified by a blue bar and labeled to the right of chromosome 2. MdBV proviral loci (L1–L8) containing proviral segments and virulence genes are identified by dark yellow bars and labeled in bold to the right of chromosomes 1, 2, and 4. Candidate, new nudivirus-like genes are identified by green bars and labeled to the left of chromosomes. Exact positions of each known nudivirus-like gene, proviral locus and proviral segment, virulence gene, and candidate, new nudivirus-like gene are presented in Tables S3, S6, S7, and S12, respectively. Color scheme for each MdBV genome component matches the color scheme used in Fig. 2 and the corresponding supplemental tables.

We used long-read PacBio sequencing and short-read Illumina sequencing to generate a new genome assembly for *M. demolitor* named iyMicdemo2.0a. A Hi-C contact map and associated summary statistics show that these data assembled into 10 chromosomes, which is substantially improved over the Mdem2 genome (Fig. 1, Table S1 and Fig. S1). The iyMicdemo2.0a assembly and previously published RNA-Seq data for *M. demolitor* (15, 19) were used with the NCBI Eukaryotic Genome Annotation Pipeline to identify a total of 13,141 genes, of which 11,885 are protein-coding (Table S2). BUSCO analysis indicated that gene annotation completeness was similar for the iyMicdemo2.0a and Mdem2 assemblies, but the former provided a global picture of where all genes reside on *M. demolitor* chromosomes that the latter could not (Table S1 and S2).

### The new assembly revises the inventory of MdBV genome components

All of the nudivirus-like genes and proviral segments that were known from the Mdem2 assembly were remapped to the new assembly (Table S3). Prediction models for the nudivirus-like genes were manually checked against RefSeq predictions and existing RNA-seq data sets to confirm whether each gene was remapped correctly and transcription start and end sites were correctly identified (Table S4). Manual annotation also revised some features of the nudivirus-like genes reported in the Mdem2 assembly. Included was the identification of MdBV *35a-10* as one gene rather than two paralogs (*35a-10-1* and *35a-10-2*), which reduced the size of the *35a* gene family from 14 to 13 members (Table S3 and S4). *odv-e66* is a single copy gene in some, but not all baculoviruses, where it encodes an envelope protein with functions in infectivity (4). All nudiviruses encode one or two *odv-e66* genes, while multiple *odv-e66* paralogs exist in *M. demolitor* and other microgastroid braconids (1, 14–17). The Mdem2 assembly identified 19 family members in *M. demolitor* (15), whereas the iyMicdemo2.0a assembly identified 23 on five chromosomes, with the length of some family members also adjusted (Table S3 and S4). Annotation of the iyMicdemo2.0a assembly identified four nudivirus-like genes in *M. demolitor* ovary RNA-seq data sets and the genomes of select other microgastroid braconids but were not annotated in the Mdem2 assembly (*p6.9-1, p6.9-2*, *DhNVorf67-like,* and *ToNVorf29-like*) (Table S3 and S4). These revisions collectively increased the number of nudivirus-like genes from 76 to 88 in the iyMicdemo2.0a assembly.

The 375 kb nudivirus cluster on *M. demolitor* chromosome 2 contained 25 of these genes: (i) 16 homologs of genes in known nudiviruses that were present in the Mdem2 assembly, (ii) *K425_459, K425_438, K425_456,* and *K425_461* also in the Mdem2 assembly, and (iii) *p6.9-1, p6.9-2,* and *ToNVorf29-like* that were previously missing or incomplete in the Mdem2 assembly annotation (Fig. 1 and Table S3 and S4). The remaining homologs of genes in known nudiviruses were elsewhere in the *M. demolitor* genome on all chromosomes, except chromosomes 3 and 8 (Fig. 1 and Table S3). Most of the nudivirus-like genes outside the nudivirus cluster were flanked by large numbers of wasp genes and distant from other nudivirus-like genes. Exceptions were the *odv-e66* paralogs on chromosomes 4, 5, and 10, and *35a* paralogs on chromosome 4, which were clustered in tandem arrays (Fig. 1 and Table S3). This pattern strongly suggested expansion of the *odv-e66* and *35a* families through duplication at several locations in the *M. demolitor* genome. Some of the other MdBV nudivirus-like genes that formed smaller families also resided in close proximity to each other, suggesting duplication (e.g., *pif-5-2* and *pif-5-3* on chromosome 6) (Fig. 1 and Table S3). As previously noted, ovary RNA-seq and RT-qPCR data classified seven of the nudivirus-like genes in the Mdem2 assembly as early, and 69 as late. The number of early genes remained unchanged in the iyMicdemo2.0a assembly, whereas the above revisions increased the number of late genes to 81 (Table S3). Some assigned names in the iyMicdemo2.0a assembly also have different aliases in the Mdem2 assembly or in the genomes of other microgastroid braconids, which we synonymize in Table S4.

In an earlier study, we generated a proteomic data set consisting of two separately collected and sucrose-gradient purified samples of MdBV virions. These samples were then further separated into envelope and nucleocapsid fractions that were used for proteomic analyses (22). Nearly all of the shared core genes that are known to encode envelope (*p74/pif-0,* most other *pif* family members) or nucleocapsid (*vp39, vlf* family members) proteins in baculoviruses were identified as envelope or nucleocapsid proteins in MdBV virions (22). Several other MdBV genes that are homologs of genes in known nudiviruses but are unknown in baculoviruses were also identified as envelope or nucleocapsid proteins (22). Since the new genome assembly revised the inventory of known nudivirus genes, we reanalyzed this proteomic data set during this study to determine if any encoded virion proteins that were not reported in our earlier analysis (22). The results identified three additional late genes that encoded envelope proteins, two that encoded nucleocapsid proteins, and two that were equivalently detected in both envelope and nucleocapsid fractions and were thus classified as virion proteins (Table S5).

The iyMicdemo2.0a assembly indicated that all proviral segments reside in eight loci as previously reported (14, 15) but also showed that misassembly of proviral segment O in the Mdem2 genome was due to tandem duplication of proviral segments G and O in locus 1 (Fig. S2). We named the two new proviral segments G1 and O1, which resulted in a total of 28 MdBV proviral segments. Locus 8 (proviral segment U) resides on *M. demolitor* chromosome 1. Locus 1 (containing proviral segments P, K, K1, Q, D, B, A, L, F, I, M, G, O, G1, and O1), locus 5 (proviral segment R), and locus 6 (proviral segment S) reside on chromosome 2 (Fig. 1). Locus 2 (proviral segments V, W, E, C, and X) resides on chromosome 4. Locus 7 (proviral segment T) resides on chromosome 6, while locus 3 (proviral segments N and J) and locus 4 (proviral segment H) reside on chromosome 7 (Fig. 1). Exact positions for each proviral locus and segment are reported in Table S6, while boundaries for each virulence gene on proviral segments and predicted protein are reported in Table S7. Manual annotation revised the lengths of some virulence genes relative to the Mdem2 assembly (Table S8). Duplication of proviral segments G and O together with other corrections also increased the total number of virulence genes from 96 in the Mdem2 assembly to 105 in iyMicdemo2.0a assembly (Table S7 and S8).

### Baculovirus and nudivirus promoter motifs do not predict MdBV early and late genes

Early studies of baculoviruses identified promoter motifs (TATAA box and an initiator sequence CA[G/T]T 100–200 bp upstream of the start codon) in some early genes that are recognized by eukaryotic RNApol II, while late genes have the invariant motif TAAG and are transcribed by baculovirus RNApol (33–35). That the baculovirus RNApol subunits (*lef-4, lef-8, lef-9,* and *p47*) are shared core genes has also led to searches for baculovirus early and late promoter motifs in nudiviruses like Heliothis zea nudivirus 1, Tipula oleracea nudivirus (ToNV1), and in some of the nudivirus-like genes in Cotesia congregata bracovirus (CcBV), MdBV, and the nudivirus-derived DEV in the parasitoid *Venturia canescens* (36–38). The results indicate that some nudivirus and nudivirus-like genes contain baculovirus promoter motifs, but many others do not. This variation could either reflect differences that are functionally important or that these motifs do not predict the RNA polymerases that transcribe them and instead vary by chance, given short lengths, low sequence complexity, and the relatively large windows that have been searched. Studies of HzNV-1 also identify ATA[G/C]G[G/C]TAT as a nudivirus candidate early gene promoter motif (18) and TTATAGTAT as a candidate late gene promoter motif (36).

Given revisions from the iyMicdemo2.0a assembly, we examined each of the 88 known nudivirus-like genes in *M. demolitor* 250 bp upstream and 50 bp downstream of the transcription start site (TSS) for the above promoter motifs. For the seven genes that transcriptome data classify as early, one (*HzNVorf128-like*) contained the two early baculovirus promoter motifs (TATAA box and an initiator sequence CA[G/T]T), two (*lef-8* and *p47*) contained a late baculovirus promoter motif (TAAG), one (*lef-4*) contained both early and late motifs, and three (*lef-9, vlf-1a,* and *lef-5*) contained none of these motifs (Table S3). A binomial test indicated that baculovirus early gene motifs did not predict the genes that expression data classify as early because the observed number (*N* = 2) significantly differed from the expectation that each would have them if they were a fully predictive marker (*P* < 0.0001). For the 81 known nudivirus-like genes that transcriptome data classify as late, 10 contained only early baculovirus promoter motifs, 34 contained only a late baculovirus promoter motif, 12 contained both, and 25 contained neither (Table S3). Again, the total number of late genes with a baculovirus late gene motif (*N* = 46) significantly differed from the expectation that all would have them (*P* < 0.0001). No MdBV early but three late genes (*35a-2, HzNVorf94-like-1,* and *27b-like*) had the second candidate nudivirus early gene motif ATA[G/C]G[G/C]TAT, while no early and only one late gene (*K425_456*) had the candidate nudivirus late gene motif TTATAGTAT (Table S3).

The 105 virulence genes on MdBV proviral segments share no homology with known nudivirus genes. As noted earlier, BV virulence genes are primarily expressed in the lepidopteran hosts that *M. demolitor* parasitizes (8, 29). As such, lepidopteran transcriptional machinery regulates virulence gene expression in hosts. Nonetheless, since these genes also reside on proviral segments and thus could also be expressed in *M. demolitor* (see below), we also examined each for the presence of baculovirus and nudivirus promoter motifs. No trends were identified with seven genes containing early baculovirus promoter motifs, 58 containing a late baculovirus motif, 11 containing both, and 29 containing neither (Table S7). Only five virulence genes contained an ATA[G/C]G[G/C]TAT or TTATAGTAT motif (Table S7). Excluding the nudivirus-like and virulence genes, we also searched all other protein-coding genes in the *M. demolitor* genome for baculovirus early and late promoter motifs within 250 bp upstream and 50 bp downstream of predicted start codons. Twenty-seven percent of these genes contained early and 65% contained late baculovirus promoter motifs. χ² tests of homogeneity detected no significant difference (*P* > 0.05) in these frequencies from the 27% of MdBV nudivirus-like genes with baculovirus early gene promoter motifs and 57% of MdBV nudivirus-like genes with baculovirus late promoter motifs. Altogether, expression data classify all of the known MdBV nudivirus-like genes as early or late, but none of the baculovirus or nudivirus promoter motifs predicted early or late gene classification. The fact that the frequency of early and late baculovirus promoter motifs was similar in the nudivirus-like versus other genes in the *M. demolitor* genome also favored the suggestion that presence varied by chance due to short length, low sequence complexity, and the search window used.

### RNAi knockdown of the MdBV RNApol downregulates the expression of most late genes

Our previous experiments showed that RNAi knockdown of MdBV RNApol subunit genes significantly downregulated transcript abundances of two MdBV late genes (*vp39* and *p74*) that nudiviruses and baculoviruses also share (21). This finding supported the fact that these subunit genes retain conserved functions as RNApol subunits, and the MdBV enzyme transcribes *vp39* and *p74*. In contrast, while MdBV *vp39* contained a baculovirus late gene promoter motif, MdBV *p74* did not in the preceding motif search (Table S3). Since baculovirus and nudivirus promoter motifs also failed to predict other MdBV early and late genes, we used an experimental approach to check if MdBV RNApol knockdown only affected MdBV late genes or it also affected other genes in the *M. demolitor* genome.

**Fig 2:**
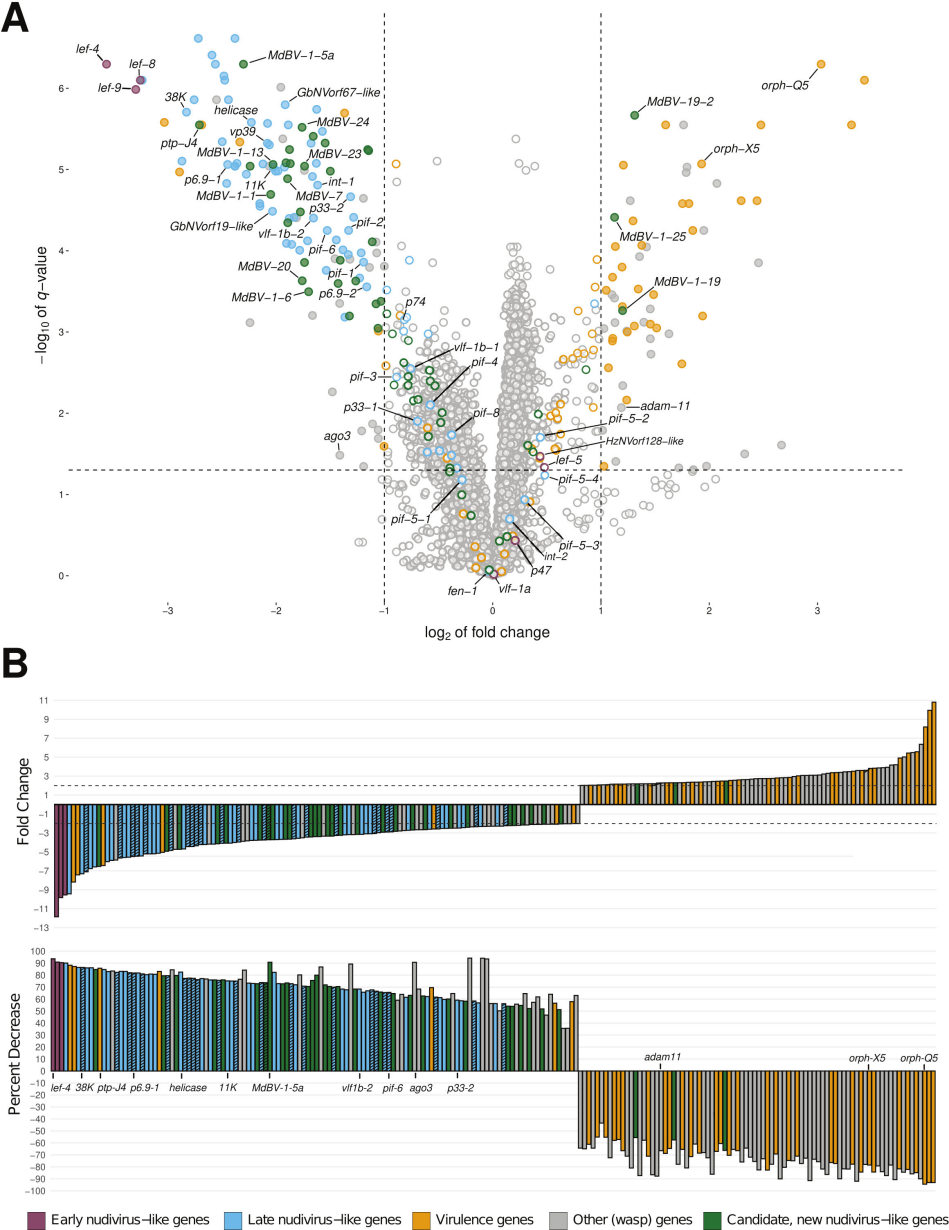
Expression of genes in the ovaries of *M. demolitor* females after RNAi knockdown of three MdBV RNApol subunit genes (*lef-4, lef-8,* and *lef-9*). (**A**) Volcano plot showing the expression of all expressed genes in ovaries from day 1 adult females treated with dsRNAs to MdBV RNApol subunit genes compared to control females treated with ds-*eGFP*. Purple circles identify the seven known early nudivirus-like genes, and blue circles identify the 81 known late nudivirus-like genes that with small exceptions, are homologs of genes in known nudiviruses. Dark yellow circles identify virulence genes that reside on proviral segments. Gray circles identify other (wasp) genes. Green circles identify the 60 candidate, new nudivirus-like genes that do not share any homology with genes in known nudiviruses. The names of some genes in each category are indicated on the volcano plot. Transcript abundance for each gene is calculated in FPKM (*N* = 6 samples per treatment). Log2 of the fold change between the two treatments is represented on the x-axis and plotted against -log10(q-value) on the y-axis. Differentially expressed genes meeting the criteria of combined significance (q-value < 0.05 and |FC| ≥ 2) are indicated by filled circles, while genes below this threshold are indicated by unfilled circles. (**B**) Fold and percent change for differentially expressed genes in the above categories are plotted from the largest to the smallest. For each downregulated gene (negative values), fold change is plotted on the y-axis by dividing the average FPKM value from the control females by the average FPKM from the treatment females. For each upregulated gene (positive values), the average FPKM value from the treatment females was divided by the average FPKM from the control females. Percent decrease in expression is also plotted on the y-axis as determined by (average control FPKM – average treatment FPKM)/average control FPKM × 100 for each downregulated gene or (average treatment FPKM – average control FPKM)/average treatment FPKM × 100 for each upregulated gene. Cross-hatched bars identify genes in the nudivirus cluster, while the solid bars indicate genes that are dispersed elsewhere in the *M. demolitor* genome.

Toward this end, we injected a cocktail of dsRNAs to three of the MdBV RNApol subunit genes (*lef-4, lef-8,* and *lef-9*) into *M. demolitor* larvae after they emerged from the host and spun a cocoon. We then isolated total RNA from the ovaries of newly emerged adult females (day 1) and conducted RT-qPCR assays, which showed that the transcript abundance of each subunit gene was reduced 90% or more when compared to control females injected with ds-*enhanced green fluorescent protein* (*eGFP*) (Fig. S3A through C). Transmission electron microscopy (TEM) showed that calyx cell nuclei from knockdown females also contained few or no MdBV virions, whereas an abundance of virions was present in calyx cell nuclei from control females (Fig. S3D and E). This latter finding was also fully consistent with previous results reporting the downregulation of *vp39* and *p74,* which encode essential capsid and envelope proteins (20, 21). We next prepared six cohorts of wasps that were injected with ds-*eGFP* and six other cohorts of wasps that were injected with our cocktail of dsRNAs that targeted RNApol subunit genes. These cohorts were then used to collect six samples of ovaries from day 1 adult control females and six samples of ovaries from treatment females that were used to prepare RNA-seq samples that we analyzed. A total of 6,755 genes were expressed in these samples, which we subdivided into the following: (i) known early nudivirus-like genes, (ii) known late nudivirus-like genes, (iii) virulence genes on MdBV proviral segments, and (iv) others which were annotated as eukaryotic (=wasp) genes (Fig. 2). A fifth category named candidate, new nudivirus-like genes shown in Fig. 2 is discussed below.

Two thresholds were used to assess differential expression of genes: q-value ≤0.05 and fold change |FC| ≥ 2 when FPKM metrics in treatment samples were compared to control samples. We used the term combined significance if a given gene exceeded both thresholds, with the significant q-value indicating that the result was not just due to random chance and the fold-change suggested that the difference was large enough to potentially affect function. This was our most stringent criterion. We used the term statistically significant if the q-value ≤0.05 but |FC| < 2. Non-significant indicated a q-value >0.05. We also noted if FPKM values for a given gene were below measurable levels to analyze. For the known early nudivirus-like genes, FPKM values for *lef-4, lef-8,* and *lef-9* were 90%–92% lower in treatment females, which mirrored our measure of knockdown by RT-qPCR (Fig. 2, Fig. S3, and Table S9). In contrast, FPKM values for the other RNApol subunits (*p47*), which were not targeted for knockdown, and the three other early genes (*lef-5, HzNVorf128-like,* and *vlf-1a*) did not significantly differ between treatment and control females (Fig. 2 and Table S9). Thus, treatment affected the RNApol subunits we targeted, but not the other early genes, which, in turn, suggested that early nudivirus-like genes are transcribed by *M. demolitor* RNApol II as found for baculovirus early genes (4, 35).

For the 81 known late genes, 25 were in the nudivirus cluster, while 56 were elsewhere in the *M. demolitor* genome (Fig. 1 and Table S3). For the nudivirus cluster, which only contains late genes, 24 were downregulated and met the threshold for combined significance after MdBV RNApol knockdown, while one (*pif-3*) did not exceed the cutoff criterion for FC but had a q-value ≤0.05 (Fig. 2 and Table S9). For the late genes outside the nudivirus cluster, 34 also exhibited combined significance and downregulation, while 14 were downregulated but classified as statistically significant because they did not exceed the cutoff criterion for FC (Fig. 2 and Table S9). Thus, an overall total of 73 late genes were downregulated after MdBV RNApol knockdown. Seven other late genes showed no difference in FC or FPKM values, while one, *odv-e66-11*, fell below measurable expression levels (Fig. 2 and Table S9). We compared transcript abundance as measured by FPKM versus RT-qPCR for one late gene in the nudivirus cluster (*vp39*) and one gene outside the nudivirus cluster (*MdBVe46*). RT-qPCR assays indicated that the transcript abundances for these genes were lower in treatment versus control females that were injected with ds-*eGFP* as found using FPKM values, which suggested that both measures yielded similar outcomes (Fig. S4).

We expected that virulence genes on MdBV proviral segments would be unaffected by knockdown of the MdBV RNApol since prior findings indicated that most are not expressed in *M. demolitor* or are expressed at very low levels, and none are homologs of known nudivirus or baculovirus genes (14, 29, 39, 40). The RNA-seq data sets from this study indicated that 34 virulence genes had average expression levels that were below measurable ranges in control females. Seventy-one had an average FPKM value of 15 in control females, but these were much lower than the average FPKM value of 571 for the known nudivirus-like genes or the average FPKM value of 79 for all other *M. demolitor* genes that were expressed in ovaries of day 1 adult females. Nonetheless, comparison of treatment to control females indicated that 39 virulence genes met our threshold for combined significance, with 32 that were upregulated and 7 that were downregulated after MdBV RNApol knockdown (Table S10). Among these genes were *orph-X5* and *orph-Q5* (upregulated) and *ptp-J4* (downregulated), which we labeled in Fig. 2. Twenty-four virulence genes were also significantly upregulated or downregulated (q-value ≤0.05) after MdBV RNApol knockdown but had |FC| values < 2, while eight showed no significant differences in FPKM or fold-change (Table S10). Some of the affected virulence genes were single copy, while others belonged to families.

For the other category containing wasp genes, 51 met our threshold for combined significance, with 23 being downregulated and 28 being upregulated after RNApol knockdown (Fig. 2 and Table S11). However, the overwhelming majority of expressed wasp genes (*N* = 6,553) did not meet our threshold for combined significance (Fig. 2 and Table S1). Downregulated genes with an average FPKM value >10 in ovaries from control females treated with ds*-eGFP* included *argonaute-2* (*ago2*) (Fig. 2 and Table S1). No upregulated genes had an average FPKM value >10 in ovaries from control samples. However, several genes with low FPKM values had FC values > 2 and a q-value of ≤0.05 when compared to treatment females. Among these was a metalloproteinase domain containing protein 11 homolog (*adam-11*) (Fig. 2 and Table S1).

### Identification of candidate, new nudivirus-like genes from the BV ancestor

**Fig 3:**
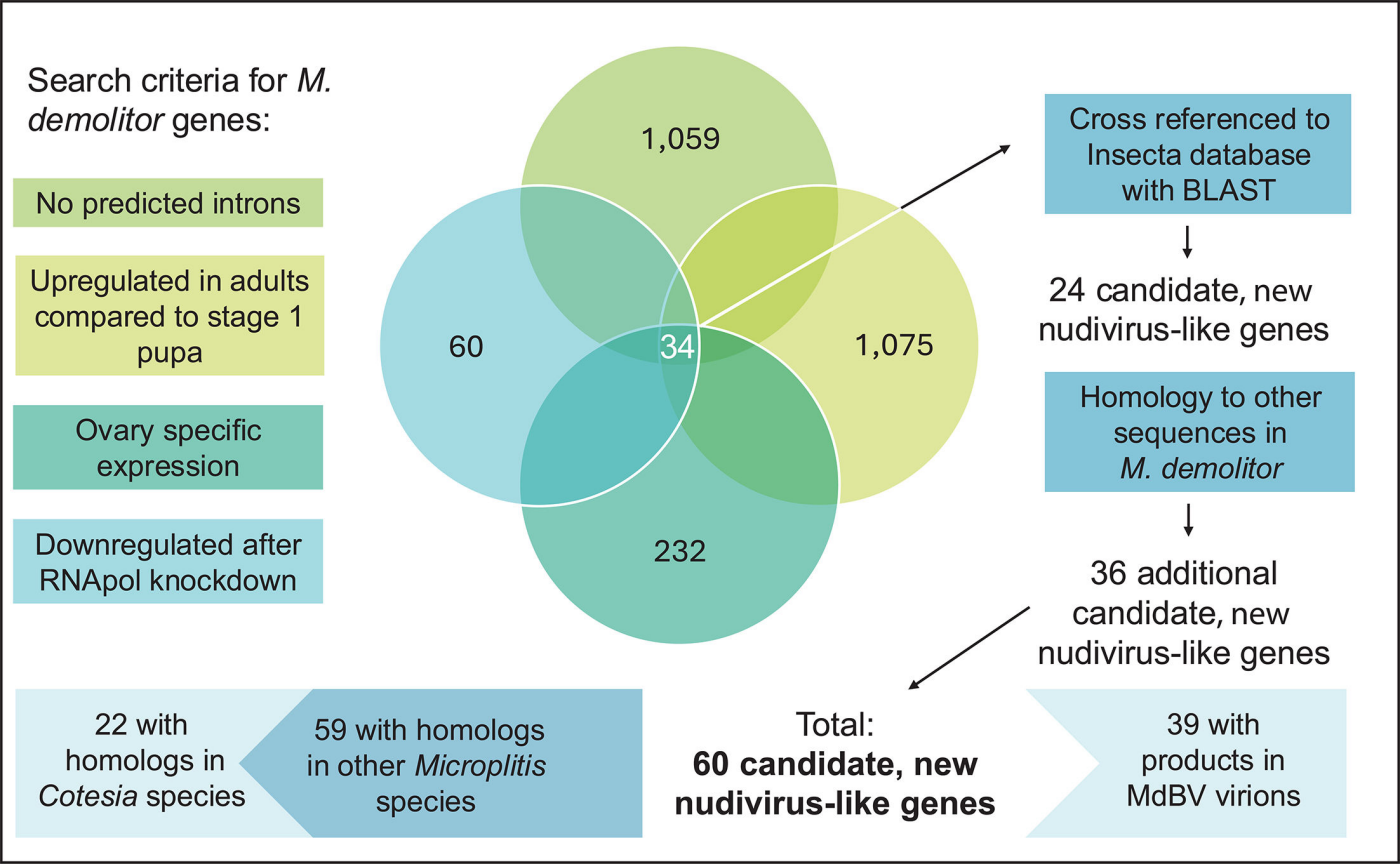
Venn diagram and flow chart showing the criteria used to identify candidate, new nudivirus-like genes from the BV ancestor in the *M. demolitor* genome. A total of 60 genes meeting all criteria were identified in *M. demolitor,* of which 39 encoded proteins detected in MdBV virions. Homologs to these genes were also identified in other *Microplitis* and *Cotesia* species.

As noted earlier, while core genes are shared among all known nudiviruses or baculoviruses, accessory genes range from being present in many, but not all species, to only being known from a single species or a small number of species within a given genus (1, 4, 31, 32). Most of the known nudivirus-like genes in BVs are either homologs of nudivirus core genes or accessory genes that are also present in known nudiviruses. However, four genes (*K425_459, K425_438, K425_456,* and *K425_461*) in *M. demolitor* and select other wasps in the subfamily Microgastrinae are absent in known nudiviruses, but their location in the nudivirus cluster together with other key features strongly suggests that they were accessory genes in the BV ancestor (14–16). These other key features are that BV nudivirus-like genes (i) are usually intronless, (ii) are specifically expressed in ovary calyx cells, (iii) mostly exhibit late gene expression profiles, and (iv) are represented by orthologs in multiple microgastroid braconid species but are unknown in other taxa. If other accessory genes from the BV ancestor exist in *M. demolitor* outside the nudivirus cluster that are absent in known nudiviruses, we reasoned most should also possess the above features. To assess this, we first noted that single-exon genes are often unannotated or incorrectly annotated by eukaryotic gene prediction pipelines. We therefore aligned reads from the ds-*eGFP*-treated RNA-seq samples of the preceding experiment to the iyMicDemo2 assembly. This identified 11,687 transcripts for proteins > 100 aa in size. We next used this data set plus RNA-seq data sets generated in previous studies (15, 19) to determine which of these transcripts mapped to predicted protein-coding genes with no introns. This identified a total of 1,093 predicted genes with no introns (Fig. 3). We then determined the total number of genes that were in the three other categories of interest, which were as follows: (i) 1,109 genes were upregulated in day 1 adult females when compared to stage 1 pupae, (ii) 266 genes that specifically or primarily were expressed in the ovaries, and (iii) 94 genes were significantly downregulated in ovaries after MdBV RNApol knockdown (Fig. 3). Excluding all of the already known nudivirus-like genes, only 34 predicted intronless genes were in each of the other three categories (Fig. 3).

We next used BLAST and the nr database for taxa in the Insecta to ask if any of the 34 genes were present outside microgastroid braconids or the small number of other parasitoids that host DEVs from other nudiviruses (Fig. 3). Ten were present in other taxa, which reduced the candidate gene list from *M. demolitor* to 24. Some of these 24 genes were single copy, while others consisted of two or more paralogs. We thus queried the iyMicDemo2 assembly by conducting additional BLAST searches with each gene to determine if additional paralogs existed. This identified 36 additional intronless genes for an overall total of 60 genes that we named as candidate, new nudivirus-like genes (Fig. 3). Names and locations of these genes in the *M. demolitor* genome are shown in Fig. 1 and listed in Table S11. Half formed a 30-member family named *MdBV-1-1* to *MdBV-1-27* (Fig. S5 and Table S11). Fifteen members of this family were tandemly arrayed on *M. demolitor* chromosome 4, while most of the others formed clusters on chromosomes 2, 6, and 10 (Fig. 1 and Table S11). An eight-member family named *MdBV-4-1* to *MdBV-4-8* clustered on chromosome 6, while *MdBV-2, MdBV-6, MdBV-8, MdBV-9, MdBV-18,* and *MdBV-19* formed two- or three-member families that were either clustered on one chromosome or dispersed on different chromosomes (Fig. 1; Fig. S6 and Table S12 ). The remaining genes (*MdBV-3, MdBV-5, MdBV-7, MdBV-10, MdBV-22, MdBV-23,* and *MdBV-24*) were single copy (Fig. 1; Fig. S7, and Table S12).

We searched the genomes of five microgastroid braconids (*Microplitis manilae, Microplitis mediator, Cotesia congregata, Cotesia glomerata,* and *Cotesia ruficrus*) in the same subfamily as *M. demolitor* (Microgastrinae) for homologs of the candidate, new nudivirus-like genes using relatively stringent criteria (percent identity >50, query coverage >60, and E-value <2 × 10^−9^). All except *MdBV-19-2* were identified in *M. manilae*; all except *MdBV-2-1, MdBV-2-2,* and *MdBV-19-2* were identified in *M. mediator,* while 22 of the candidate, new nudivirus-like genes in *M. demolitor* had homologs in one or more of the *Cotesia* species (Fig. 3 and Table S13). We next searched the genomes of *Chelonus insularis* and *Chelonus formosanus,* which are in the evolutionarily most distant subfamily (Cheloninae) in the microgastroid complex from the Microgastrinae (11, 13, 17). None of the candidate, new nudivirus-like genes were present in either species using the above criteria, but relaxing the percent identity criterion to >40% identified a single *MdBV-11* homolog in both *Chelonus* species. We also examined the genomes of two braconids (*Fopius arisanus* and *Diachasmimorpha longicaudata*) outside the microgastroid complex, which were selected because *F. arisanus* contains a DEV from a different nudivirus, while *D. longicaudata* contains a poxvirus (Poxviridae) that has also been coopted for functions in parasitizing hosts (41, 42). Neither contained genes with any homology to the candidate, new nudivirus-like genes in *M. demolitor*.

### Thirty-nine of the candidate, new nudivirus-like genes encode proteins in MdBV virions

All 60 candidate, new nudivirus-like genes in *M. demolitor* were classified as late because they were upregulated in ovaries from day 1 adults when compared to Stage 1 pupae (Table S12). Similar to the nudivirus-like genes that had been identified prior to this study, some of the candidate, new nudivirus-like genes contained baculovirus early or late promoter motifs, but none contained the early or late promoter motifs described from HzNV-1 (Table S12). We thus analyzed the effect of MdBV RNApol knockdown on these candidate, new nudivirus-like genes as earlier done for the known nudivirus-like genes. The results showed that 29 of 60 (48%) were downregulated by knockdown of the viral RNApol and met our threshold for combined significance (Fig. 2 and Table S14). Sixteen others were also downregulated and had significant q-values, but FC values were <2 (Table S14).

As noted earlier, we previously generated proteome data sets for MdBV virions that were also separated into envelope and nucleocapsid fractions (22). Reanalyzing these data sets indicated that 39 of the candidate, new nudivirus-like genes encoded proteins in MdBV virions, while 21 did not (Table S15). Among the 39 genes, 15 were classified to encode envelope (E) proteins because matching unique peptide sequences were significantly more abundant in the envelope fraction (Table S15). Three were classified as nucleocapsid (N) proteins, while 21 were classified as virion (E/N) proteins because unique peptide abundances did not significantly differ between the envelope and nucleocapsid fractions (Table S15). For each of the candidate, new nudivirus-like genes, the total number of unique peptide counts was similar to the range of unique peptide counts for the core gene products MdBV shares with baculoviruses and/or nudiviruses we previously identified (22). For example, abundant envelope and nucleocapsid proteins in baculoviruses include P74/Pif-0 and Vp39, respectively, while proteins like Pif-6 are less abundant (8, 43). Correspondingly, prior results identified 73 unique peptides in the envelope fraction that corresponded to MdBV P74/Pif-0, 31 unique peptides in the nucleocapsid fraction that corresponded to MdBV Vp39, but only 9 unique peptides that corresponded to Pif-6 (22). The range of unique peptide counts for the candidate, new nudivirus-like genes shown in Table S15 ranged from a high of 161 in the envelope fraction of MdBV-22 to a low of 3 in the nucleocapsid fraction for MdBV-1-14. Thus, the range of unique peptide counts for the candidate, new nudivirus gene products detected in virions were broadly similar to previously published results that focused on already known nudivirus genes (22), and the few additional known nudivirus genes that were identified to encode virion proteins during this study (Table S1).

**Fig 4:**
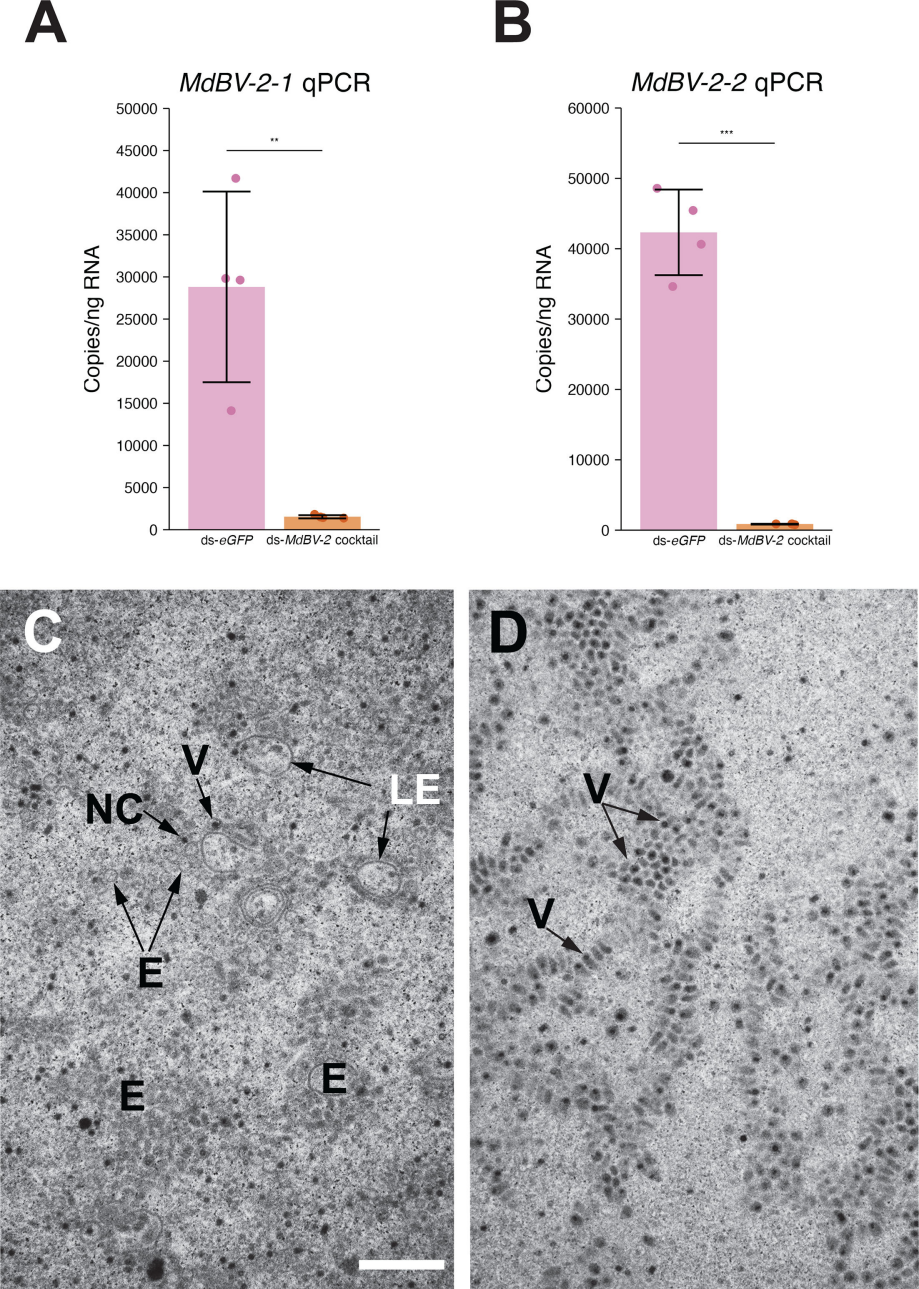
RNAi knockdown of *MdBV-2-1* and *MdBV-2-2* disables MdBV virion formation. (**A**) Transcript abundance of *MdBV-2-1* per nanogram of total RNA in ovaries from day 1 adult females injected with a cocktail of ds-*MdBV-2-1* and ds-*MdBV-2-2* or ds-*eGFP* (control) as Stage 1 pupae. Bars show mean transcript copy ± standard error, solid circles indicate the number of females examined, and asterisks indicate that treatment significantly differed (two-tailed *t*-test, **, *P* < 0.01). (**B**) Transcript abundance of *MdBV-2-2* with treatments, bars, sample sizes, and statistical analysis as defined in A (two-tailed *t*-test, ***, *P* < 0.001). (**C**) TEM image of a calyx cell nucleus from a day 1 adult female after *MdBV-2-1* and *MdBV-2-2* knockdown. The nucleus contains an abundance of MdBV envelopes (E) and small numbers of electron-dense nucleocapsids (NC) that are usually not surrounded by an envelope. Arrows point to presumptive newly formed envelopes, while envelopes in clusters contain light gray material, but most contain no electron-dense nucleocapsids. Several large envelopes (LE) are also present, which, in some cases, surround MdBV envelopes and nucleocapsids. (**D**) TEM image of a calyx cell nucleus from a day 1 adult control female treated with ds-*eGFP*. The nucleus contains primarily mature MdBV virions (V), which consist of an envelope containing light gray material and one electron-dense nucleocapsid. Comparing the images in C and D to one another clearly indicates that *MdBV-2-1* and *MdBV-2-2* knockdown substantially reduces the number of mature virions that are present when compared to the control. Scale bar in C equals 1 µm with magnification the same in D.

We selected four of the genes encoding MdBV virion proteins for further study, *MdBV-2-2* and *MdBV-11,* which were classified to encode virion components, and *MdBV-5,* which encoded a predicted envelope protein (Table S15). Since *MdBV-2-2* shares extensive sequence similarity with *MdBV-2-1,* which was not detected in virions (Fig. S6), we injected a cocktail of specific dsRNAs that targeted both paralogs into Stage 1 pupae, followed by RT-qPCR assays and TEM analysis of calyx cells from day 1 adults (Fig. 4). The results showed that *MdBV-2-1* and *MdBV-2-2* were both knocked down more than 90% when compared to control females that were injected with ds-*eGFP* (Fig. 4A and B). TEM analysis indicated that *MdBV-2-1/MdBV-2-2* knockdown substantially reduced the number of mature virions, which are normally characterized by an envelope surrounding a single, electron-dense nucleocapsid (Fig. 4C). Instead, *MdBV-2-1/MdBV-2-2* knockdown was associated with calyx cell nuclei in day 1 adult females containing an abundance of envelopes and a reduced number of electron-dense nucleocapsids that usually lacked an envelope (Fig. 4C). This phenotype differed from day 1 adult control females that were treated with ds-*eGFP* where calyx cell nuclei predominantly contain mature virions (Fig. 4D). *MdBV-2-1/MdBV-2-2* knockdown was also associated with the presence of large envelopes in calyx cell nuclei that sometimes contained smaller virion envelopes and/or nucleocapsids (Fig. 4C). The collective effects of *MdBV-2-1/MdBV-2-2* knockdown could overall suggest a delay in the timing of virion assembly since prior studies show that early in virion morphogenesis envelopes initially appear followed by electron-dense capsids that are then individually surrounded by an envelope (20–22). However, inspection of calyx cells from multiple females indicated that *MdBV-2-1/MdBV-2-2* knockdown resulted in a persistent reduction in the number of mature virions that assembled in *M. demolitor* calyx cells, suggesting that the primary defects were a reduction in the number of nucleocapsids that formed and envelopes not surrounding most nucleocapsids. For *MdBV-11* and *MdBV-5,* RT-qPCR assays indicated that both were knocked down more than 90% in day 1 adult females, TEM analysis of calyx cell nuclei showed no phenotypic alterations in the morphology or abundance of virions when compared to control females (Fig. S8).

### A candidate promoter motif associated with MdBV late genes

**Fig 5:**
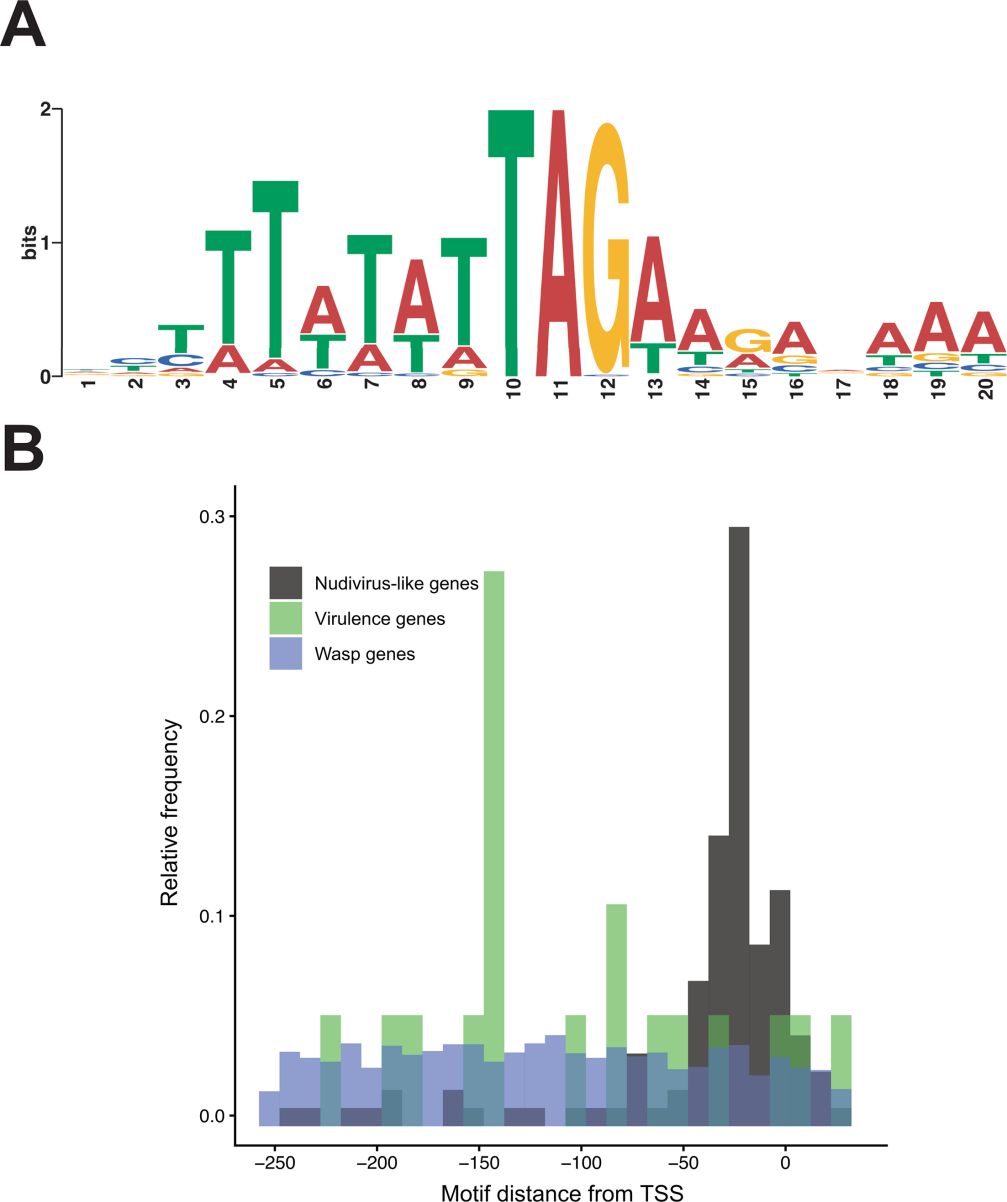
Analysis of the MdBV late motif identified in the 85 late nudivirus-like genes that were downregulated by MdBV RNApol knockdown. (**A**) Sequence logo of the motif generated by MEME software. (**B**) Location of the MdBV late motif from the TSS. Bars indicate the relative frequency of nudivirus-like genes (gray), virulence genes (green), or wasp genes (blue) with the late motif at a given distance from the TSS. Only genes with a late motif within 250 bp upstream or 50 bp downstream of the TSS are plotted.

The total number of nudivirus-like genes in the *M. demolitor* genome increased to 148 with the 60 new candidates. The preceding results further classified 141 of these as late genes, of which 118 (84%) were downregulated by MdBV RNApol knockdown. Given baculovirus and nudivirus promoter motifs were inconsistently present in these downregulated genes, we reexamined each by inputting sequences 250 bp upstream and 50 bp downstream of the TSS into MEME to determine if any shared motif existed. The results identified a 20 bp sequence in 85 of the 118 (72%) downregulated genes, with an E-value of 1.0e^−29^ (Fig. 5A and Fig. S9). We named this the MdBV late motif, which was AT rich and had an invariant TAG sequence that was similar to the baculovirus late gene motif TAAG (Fig. 5A). We used a nucleotide frequency model with a *P*-value cutoff of 0.001 to assess the total number of genes in the *M. demolitor* genome with an MdBV late motif from 250 bp upstream to 50 bp downstream of the predicted TSS. Twenty percent of wasp genes, 17% of virulence genes on proviral segments, and 74% of all nudivirus-like genes had a late MdBV motif (Table S16; each nudivirus-like and virulence gene with this motif is listed in Tables S3, S7, and S12). A binomial test rejected the hypothesis that the MdBV late motif existed in proportion to the number of genes in each category (χ² = 210.8; df = 2; *P* < 0.0001), which indicated more nudivirus-like genes had this motif than expected by chance, while fewer wasp and virulence genes did. We additionally examined if the MdBV late motif was differentially present in virulence genes that were differentially expressed after knockdown of the MdBV RNApol versus virulence genes that were not differentially expressed or had FPKM values that were below measurable ranges in control females. The results showed that the MdBV late motif was present in 22% of the former (11 of 62), and 17% of the latter (7 of 41). Contingency table analysis indicated that these frequency distributions did not significantly differ from one another, which supported the conclusion that the presence of a MdBV late gene motif was not predictive for differential expression in response to MdBV RNApol knockdown (χ² = 0.008; df = 1; *P* = 0.93). Analysis of the *C. congregata* genome, which harbors CcBV, showed the same pattern as *M. demolitor* (Table S17), whereas the proportion of genes with a late MdBV motif in three nudiviruses and one baculovirus was lower than that in the nudivirus-like genes in *M. demolitor* or *C. congregata* (Table S18). TSSs of baculovirus late genes usually reside within a few nucleotides of the TAAG motif (33–35). The MdBV late motif was similarly very near, 20–30 bp upstream, of the TSS in most nudivirus-like late genes (Fig. 5B). In contrast, position trended further upstream in the virulence genes that had a late motif, while position in wasp genes showed no trend in the window upstream and downstream of predicted TSSs that we searched (Fig. 5B).

## DISCUSSION

This study advances our understanding of BVs by presenting a chromosome-level genome assembly for *M. demolitor,* globally assessing the effects of MdBV RNApol knockdown on gene expression in the ovaries, and using several shared features to identify candidate, new nudivirus-like genes from the BV ancestor that share no homology with genes in known nudiviruses. The new assembly corrects errors and identifies the location of all nudvirus-like genes and proviral segments in the *M. demolitor* genome. The new assembly also greatly facilitated study of the genome-wide effects of MdBV RNApol knockdown and discovery of the candidate, new nudivirus-like genes we report.

*C. congregata* provides the only other chromosome-level genome assembly for a microgastroid braconid in the published literature (16). *C. congregata* and *M. demolitor* are both in the subfamily Microgastrinae but last shared a common ancestor 53 million years ago, indicating long-term divergence (11, 13). Genome-wide studies of insects and other animals generally show that large-scale gene content and order over chromosomes (macrosynteny) usually decays more rapidly than local clusters (microsynteny), where selection can preserve content and order of functionally related genes (44–47). Comparing *M. demolitor* and *C. congregata* suggests these trends apply to MdBV and CcBV where broad-scale rearrangements result in little macrosynteny in terms of where nudivirus-like genes and proviral loci are located in each wasp species. As one example, the nudivirus cluster in *M. demolitor* resides on chromosome 2, which contains three proviral loci (numbered 1, 5, and 6) and two *odv-e66* family members, whereas the nudivirus cluster in *C. congregata* resides on chromosome 7, which contains no proviral loci or *odv-e66* family members (16). On the other hand, several similarities in microsynteny exist. One of these is the nudivirus cluster itself where gene content and order are near-fully conserved in *M. demolitor* and *C. congregata* (14). Experimental studies also show that several of the late genes in the nudivirus cluster of each species are essential for virion formation (20–22). Proviral loci and segments in *C. congregata* and *M. demolitor* also share orthology relationships. These include that two loci in each species contain most of the proviral segments. Proviral loci also share conserved flanking motifs with functions in amplification, while proviral segments within loci also share flanking motifs with functions in producing the circular dsDNAs that are packaged into capsids (14, 23, 24). The Cheloninae is the most distantly related subfamily in the Microgastroidea from the Microgastrinae (17, 48). Chelonine parasitoids produce virions in calyx cell nuclei that are morphologically similar to MdBV and CcBV virions, but sequencing identified little synteny between *C. insularis* bracovirus (CiBV) and MdBV or CcBV genome components (17, 48). For example, only three small clusters of nudivirus-like genes in *C. insularis* share syntenic order with the nudivirus cluster of MdBV and CcBV (14, 16, 17). This finding further suggests that overall microsynteny of the nudivirus cluster in *M. demolitor* and *C. congregata* is not essential for virion formation, although the order of certain genes in the nudivirus cluster might be. Microsynteny of genes surrounding proviral loci suggests few similarities between *C. insularis* and *M. demolitor* (17). Motifs in *M. demolitor* and *C. congregata* that identify the amplification boundaries of proviral loci are also not conserved in *C. insularis* (17, 20, 21). All BVs lack a nudivirus or baculovirus-like DNA polymerase, which suggests that this shared core gene was lost early in BV evolution (14, 16, 17). Thus, amplification of proviral segments is thought to depend on a still unknown wasp DNA polymerase, but the motifs this enzyme recognizes appear to differ in microgastrines like *M. demolitor* and *C. congregata* and chelonines like *C. insularis*. In contrast, the motifs that identify processing sites for proviral segments are conserved between MdBV, CcBV, and CiBV, which is consistent with each retaining the nudivirus-like integrases that have been shown to process MdBV proviral segments (14, 17, 20, 21, 23).

We began the second part of the study by finding that baculovirus early and late gene motifs were variably present in MdBV nudivirus-like genes. This result was consistent with baculovirus promoter motifs also being variably present in genes of known nudiviruses, nudivirus-like genes in other BVs, and the nudivirus-like genes of the *V. canescens* DEV (14, 36–38, 49). That similar proportions of *M. demolitor* genes contain baculovirus motifs further argued against these sequences being key features of MdBV nudivirus-like genes, which also largely lacked the candidate early and late promoter motifs described from the betanudivirus HzNV-1 (18, 36). In contrast, our RNAi assays very strongly indicate that knockdown of the MdBV RNApol downregulates most of the known nudivirus-like late genes while having no effect on the nudivirus-like early genes or the vast majority of wasp genes.

Prior studies show that proviral segments of all studied BVs encode virulence genes, which, in some cases, are single copy, while in others, they have diversified into multimember families of varying size (6, 8, 10). The total inventory of virulence genes also varies among the BVs that are produced by wasps in different subfamilies or individual species. This variation is known to be driven by a combination of gene acquisition from different sources and gene family diversification after acquisition (10, 50). Known sources of virulence genes include both the translocation of wasp genes into proviral segments and horizontal transfer from other organisms (10, 50). None of the virulence genes in BVs share significant homology with genes in known nudiviruses, but it is possible that some virulence genes originated from the BV ancestor, given the findings reported previously and in this study, indicating that accessory genes absent in known nudiviruses were also likely inherited from the BV ancestor (14, 16). The results from this study show that MdBV RNApol knockdown altered transcript abundance of some virulence genes in *M. demolitor* ovaries when compared to control females treated with ds-*egfp*. However, we are uncertain about the biological significance of this finding because FPKM values for the virulence genes are very low when compared to the nudivirus-like genes that are expressed in *M. demolitor* ovaries. Virulence gene FPKM values are also very low when compared to prior results showing that many of these genes are expressed at much higher levels in parasitized hosts where several studies also show that some of these genes have functions that are essential for the successful development of *M. demolitor* offspring (26–30). BV virulence genes are presumably transcribed in hosts by RNApol II since none of the subunit genes that produce MdBV RNApol are present in the virions that females inject into hosts (6, 10, 15). In contrast, most virulence genes lacked the MdBV late gene promoter motif that most of the nudivirus late genes possess that were downregulated by MdBV RNApol knockdown. Thus, differential expression of some virulence genes after MdBV RNApol knockdown could be an indirect response to other alterations that occur in calyx cells in association with the downregulation of most nudivirus-like late genes. Current evidence also does not identify any functions for virulence genes in ovary calyx cells of *M. demolitor*. On the other hand, another study recently reports that some BV virulence genes in another microgastrine braconid (*Cotesia typhae*) are overexpressed in pupal stage ovaries when compared to adult-stage ovaries (51). Thus, stage-specific differences in the expression of BV virulence genes in wasps may more commonly occur than is generally recognized. It is also possible some virulence gene products could be produced during virion morphogenesis and potentially introduced into hosts as a means to providing short-term functions that benefit wasp offspring before virulence gene products are transcribed and translated in host cells that virions infect. However, inspection of our MdBV virion proteomic data sets does not identify the presence of any virulence gene products that we know from prior studies are expressed in parasitized hosts and have functions that benefit *M. demolitor* offspring (8, 10, 26–30).

Dispersal of genes from the BV ancestor throughout the genomes of wasps makes the identification of nudivirus-like genes very difficult in the absence of homology to genes in known nudiviruses. We used sensitivity to MdBV RNApol knockdown together with other traits that known BV nudivirus-like genes share to identify 60 other candidate, new nudivirus-like genes in *M. demolitor*. Evidence that these genes originate from the BV ancestor is strongest for the 39 that encode proteins detected in MdBV virions. For the 21 other genes, 14 also likely derive from the BV ancestor because they are in the same families (i.e., paralogs) as the genes whose products were detected in virions. The other seven may have functions in virion formation but are not related to genes that were detected to encode virion components. Conducting functional assays on all 60 of the candidate new nudivirus-like was beyond the scope of this investigation. However, we chose four for further study as proof of principle that some are functionally important for virion formation by assessing the effects of knocking down each by RNAi. The genes we targeted were single copy or formed a two-member family that we were confident we could knock down. Our results strongly support that knockdown of *MdBV-2-1* and *MdBV-2-2* compromises virion formation. We did not determine if this effect was solely due to *MdBV-2-2,* which encodes a protein detected in virions versus it also involves *MdBV-2-1,* which encodes a closely related but not identical protein that was not detected in virions. That no alterations in virion formation were associated with RNAi knockdown of *MdBV-5* or *MdBV-11* suggests these genes encode proteins that are detected in virions, but neither is essential for envelope formation, capsid formation, or virion assembly. However, we also note that MdBV-11 was only detected in one versus both of our nucleocapsid samples, which could mean that it is not a consistent nucleocapsid component and thus did not affect virion morphology or morphogenesis when knocked down by RNAi.

Our search for homologs of the candidate, new nudivirus-like genes showed that nearly all were present in two other *Microplitis* species, 22 were present in other *Cotesia* species, and none were present in the two *Chelonus* species we examined. In comparison, wasps in these genera retain the same core genes nudiviruses share with baculoviruses, whereas other known nudivirus-like genes such as *HzNVorf94-like, K425_438, K425_456, K425_461, int-2, fen-1, PmNVorf87-like*, and *ToNVorf54-like* that *M. demolitor* and *C. congregata* share have been lost in *Chelonus insularis* (14–17). Thus, core genes shared with nudiviruses and baculoviruses are likely retained by all BVs, while accessory genes show evidence of being variably retained, lost, or are difficult to detect with increased evolutionary distance. Alternatively, the above differences along with none of the 60 new nudivirus-like genes we identified in *M. demolitor* being present in the two *Chelonus* genomes could suggest that the BVs in the subfamily Cheloninae derive from endogenization of a nudivirus that differs from the nudivirus that endogenized into the common ancestor of the subfamily Microgastrinae that *Microplitis* and *Cotesia* spp. belong to. Distinguishing between these possibilities will require more comparative data of BVs among the subfamilies of the microgastroid complex than is currently available. However, the approaches used in this study to identify the candidate new nudivirus-like genes in *M. demolitor* could be used to identify nudivirus-like accessory genes from parasitoids in different subfamilies or genera of microgastroids, which could help address whether BVs more likely derive from a single nudivirus ancestor versus multiple nudivirus ancestors.

The main conclusion from our genome-wide knockdown experiments is that the MdBV RNApol transcribes most of the nudivirus-like late genes but shows little evidence for being coopted to transcribe the large number of wasp genes that are also specifically expressed in ovaries when virions are being produced. This finding is also consistent with the conclusion that the primary function of the BV RNApol remains conserved, given evidence that the primary function of the baculovirus RNApol is also to transcribe late genes, and inferential evidence that the same is the case for nudiviruses (4, 18, 33–35). However, results from this study and other comparative data also suggest that late gene promoter motifs differ between baculoviruses and nudiviruses and have also changed between nudiviruses and nudivirus-like genes in BVs and other nudivirus-derived DEVs (14, 18, 35, 38, 49). We thus ended this study by asking if MdBV nudivirus-like genes that our experimental studies showed to be downregulated by knockdown of the MdBV RNApol potentially share sequences near the TSS that would be consistent with being a BV late gene motif. We present evidence that a 20 bp motif in most downregulated late genes has features that are consistent with being a motif that BV RNApols recognize. We earlier noted that the invariant TAG in the center of this motif is similar, but not identical to the TAAG sequence that baculovirus late genes share (4, 35). Interestingly, a number of late genes for the nudivirus-derived DEV in *V. canescens* also has an upstream motif containing a near-invariant TAG sequence (38). Among the nudivirus-like late genes that were unaffected by MdBV RNApol knockdown, three (*pif-5-1, pif-5-3,* and *pif-5-*4) are paralogs of a five-member *pif-5* family in *M. demolitor* whose proteins are not detected in MdBV envelopes (22). A fourth nudivirus-like gene, *int-2,* that was unaffected by MdBV RNApol knockdown also produces a protein that is not detected in virions or required for proviral segment processing as found for other tyrosine recombinase family members such as *int-1* (20). Thus, most of the late genes unaffected by MdBV RNApol knockdown show evidence of producing proteins that no longer have functions in MdBV virion formation and may be evolving neutrally.

In summary, the new genome assembly for *M. demolitor,* together with knockdown assays and differential expression analysis, indicates the MdBV RNApol primarily transcribes nudivirus-like late genes. Our results also identify several genes in *M. demolitor* that also likely derive from the BV ancestor. Future studies will be needed to understand the processes that upregulate the RNApol subunits and other early nudivirus-like genes in calyx cells after females pupate. In addition, while RNAi-based experiments provide insights into the functions of some of the nudivirus-like late genes in microgastroid braconids, others, including most of the new genes reported in this study, require future investigation. Finally, genome components in ancient DEVs like BVs are spread throughout wasp genomes, which makes the identification of genes from the ancestor virus very difficult if they lack homology to known nudiviruses. In this respect, the approach used here to identify candidate, new nudivirus-like genes was very helpful. In contrast, the same approaches may be less useful in more recently derived DEVs, for which genes are mostly located in clusters and/or are less divergent from the virus ancestor, as is the case for the nudivirus-derived DEV in *Fopius* wasps or the DEV in *V. canescens* (41, 52–54).

## MATERIALS AND METHODS

### Insect rearing

*M. demolitor* was reared as previously described by parasitizing larval-stage *Chrysodeixis includens* (Lepidoptera: Noctuidae) (55). In brief, all life stages of each insect were maintained at 27°C with a 16 h-light:8 h-dark photoperiod. *C. includens* larvae were reared by feeding them an artificial diet (Southland Products). Adults were held in plastic containers with a 10% sucrose solution. Adult *M. demolitor* females were allowed to parasitize *C. includens* second or third instars. The resulting wasp offspring developed for 6 days in *C. includens* larvae that were fed artificial diet. A single wasp emerged per host and spun a cocoon 7 days post-parasitism, followed by pupation. Adult wasps emerged 4 days later and were placed in plastic cages where they were provided *ad libitum* access to water and honey.

### Genome sequencing

A PacBio high-fidelity (HiFi) library was prepared from a single adult male *M. demolitor* wasp that was cryogenically ground with a Spex GenoGrinder 2010 before the extraction of high molecular weight DNA using the Qiagen MagAttract HMW DNA kit. The library was prepared using the Express Template Prep Kit 2.0 following the PacBio low-input HiFi protocol and optional nuclease digestion step after preparation. The library was size selected with 40% diluted AMPure BP beads to remove library molecules < 3 kbp as outlined in the PacBio library preparation protocol. DNA quality, quantity, and purity were assessed using a Qubit HS DNA kit and Fragment Analyzer. Libraries were prepared using the Sequel II Binding Kit 2.0 and sequenced on a PacBio Sequel II with a 30 h run time.

A Hi-C library was also prepared from four pooled adult male wasps from the same mother as the single male wasp used for HiFi library preparation. These wasps were ground in liquid nitrogen and placed in freshly prepared fixation buffer using the low-input crosslinking protocol for the Arima-HiC 2.0 kit. The Arima-HiC 2.0 kit was used to perform proximity ligation on the cross-linked sample. Proximity ligated DNA was then sheared using a Diagenode Bioruptor. Sheared DNA was size selected for 200–600 bp DNAs using a two-sided bead clean-up, followed by checking size distribution using an Agilent TapeStation. An Illumina library was prepared using the Swift Accel-NGS 2S Plus DNA library kit per the Arima-HiC 2.0 protocol. The library was single indexed, and amplification was performed using a KAPA HiFi HotStart 2× Master Mix and 10× Illumina Amplification primers with eight cycles of PCR. Quality control steps were performed with the Qubit HS DNA kit and Agilent TapeStation before sequencing on an Illumina iSeq. Hi-C library preparation generated 5,547,384 reads.

PacBio HiFi reads (SRA accession number: SRR20458489) were filtered using HiFiAdapterFilt (v3.0.1) to remove reads containing residual PacBio adapter sequences (56). Filtered HiFi reads were assembled using HiFiASM-0.13 (57). Hi-C reads (SRA accession number: SRR20639500) were aligned to the genome using BWA-MEM2 (v2.2.1) (58), and Phase Genomics tools (https://github.com/phasegenomics/juicebox_scripts) were utilized to generate Hi-C files for the alignment prior to manually scaffolded assembly of the Hi-C data using Juicebox (v2.17.00) (59).

### Genome annotation

Genome annotation of the chromosomal-level *M. demolitor* assembly was performed using the NCBI Eukaryotic Genome Annotation Pipeline (https://www.ncbi.nlm.nih.gov/refseq/annotation_euk/process/) using the reference genome produced in this study and previously published *M. demolitor* RNA-seq data in GenBank (BioProject: PRJNA214515) (14, 15). Further statistics and annotation evidence can be found at https://www.ncbi.nlm.nih.gov/refseq/annotation_euk/Microplitis_demolitor/GCF_026212275.2-RS_2023_02/. Gene annotation completeness was analyzed with BUSCO v4.1.4 using the hymenoptera_odb10 lineage data set (60).

### MdBV gene annotation

Known MdBV genes from the Mdem2 assembly and annotation micdem_OGSv1.0 (https://doi.org/10.15482/USDA.ADC/1521095) were remapped to the new assembly via LiftOff software v1.6.3 (https://github.com/agshumate/Liftoff/releases/tag/1.6.3), using the -exclude_partial and -polish options. These data were viewed as a track along with the NCBI RefSeq predictions described above and were manually compared in the *M. demolitor* jBrowse/Apollo viewer in the i5K workspace (https://i5k.nal.usda.gov/available-genome-browsers). Lifted over MdBV gene models were evaluated against RefSeq predictions and existing RNA-seq data sets to determine if the genes had been correctly annotated. Genes were edited to adjust transcription start and end site boundaries corresponding to RNA-seq sequence read coverage from *M. demolitor* ovaries (19) and to remove incorrect introns when necessary. BLASTP was used to locate additional MdBV genes identified in related wasp genomes but not annotated in the previous *M. demolitor* v1.0 OGS (15, 61). Several gene predictions neighboring known viral genes in the *odv-e66* gene family were evaluated with BLASTP and aligned with Muscle (v5.1.0) before being annotated as additional *odv-e66* family members (61, 62). The great majority of remapped annotations from the previous genome assembly needed no altering. A small number of previously unannotated MdBV genes were identified and added to this OGS: *DhNVorf67-like, ToNVorf29-like,* and *p6.9-1* and *-2,* as well as *odv-e66-22* through *-27*, while *odv-e66-9*, *-21*, and *-24* were removed because they were located on previously misassembled, duplicated sequences that were resolved in the new assembly. In the manual annotation process, gene names were also altered to reflect the most up-to-date nomenclature in the literature. As part of the annotation process, we additionally assessed whether any of the previously unannotated MdBV genes encoded virion components by reanalyzing a previously generated proteomic data set for MdBV virion envelopes and nucleocapsids (see details on methods below) (22). Manual annotations were merged with NCBI annotations to create the *Microplitis demolitor* Official Gene Set (OGS) v2.0 (https://doi.org/10.15482/USDA.ADC/28590425). MdBV gene cluster amplification boundaries were also defined using previously described sequence motifs (24, https://doi.org/10.15482/USDA.ADC/28590425).

### Proviral segment annotation

MdBV proviral segments can be identified in the *M. demolitor* genome by examining sequence coverage from sequencing the DNAs that are packaged into MdBV virions, which have short but conserved excision motifs at each end (14, 15). Illumina sequence reads from MdBV were mapped to the genome using BWA (v0.7.17) (58), and a bigwig track was generated with DeepTools (v3.5.2) (14, 15, 63). Proviral segment locations in the iyMicDemo2.1a assembly were identified using BLASTN (v2.13.0) of the proviral segment queries against a database containing the reference genome with default parameters (61). Proviral segment boundaries were manually identified as previously reported using coverage information from bigwig tracks displayed within the jBrowse instance of the genome hosted on the i5k website (https://i5k.nal.usda.gov/available-genome-browsers), and the presence of sequence motifs known to flank proviral segments as previously outlined (15, 24). Virulence genes on proviral segments were remapped from the previous to the new genome assembly as described above. The remapped genes were manually checked using RNA-seq data from *C. includens* caterpillars infected with MdBV (PRJNA285771/SAMN03758721; PRJNA437008/SAMN08637637; SAMN08637639; SAMN08637643-SAMN08637645) and edited as described above, as well as ensuring that gene boundaries fell within proviral segments using the *M. demolitor* jBrowse/Apollo viewer in the i5K workspace (15, 24, 29). In the previous assembly, proviral Segment O could not be assembled correctly, preventing the annotation of genes in the *glc* and *egf* families belonging to this segment (15, 40). These genes, and any other newly annotated genes on proviral segments, were added using BLASTP (v2.13.0) to search their sequences from proviral segment sequencing against the new assembly and named according to their homology to known virulence genes (61). These manual annotations were also included in the merge with NCBI annotations described above to create the *Microplitis demolitor* Official Gene Set (OGS) v2.0.

### Gene knockdown assays

Three MdBV RNApol subunit genes (*lef-4, lef-8,* and *lef-9*) were knocked down by RNA interference using previously established methods (20, 21, 64). In brief, dsRNAs were made with the Ambion MegaScript RNAi kit using cDNA from adult wasp ovaries amplified with gene-specific primers designed with an added 5′ T7 promoter adapter to amplify a 300–400 bp template with standard PCR (Table S19). dsRNAs to *lef-4, lef-8,* and *lef-9* were combined to create a dsRNA cocktail with a total concentration of ~ 500 ng/µL. Control treatment dsRNA was synthesized for a non-specific dsRNA probe eGFP and diluted to 500 ng/µL. From 0.5 to 1.0 µL of either the dsRNA cocktail or ds-*eGFP* was individually injected into larval-stage wasp abdomens within 15 h of cocoon spinning. Within 24 h of adult emergence, ovaries were explanted from female wasps into 1× phosphate-buffered saline (PBS).

Total RNA was extracted from each set of ovaries using the Zymo QuickRNA Mini-Prep kit and two DNase digestion steps: one performed on-column within the kit standard protocol and a second with a TURBO DNA-free kit, followed by sodium acetate precipitation for RNA clean-up. Gene knockdowns were measured using RT-qPCR assays performed with cDNA synthesized from a normalized amount of RNA from each sample and 100–200 bp primers targeting each gene (Table S20). Within an experiment, a consistent amount ranging between 25 and 100 ng of total RNA was used for each cDNA reaction. Absolute standard curves for each gene were generated from serial dilutions (10^2^–10^7^) with known copy numbers of a synthesized gBlock (IDT) of target amplicons with 10 bp spacers between primer target sequences (Table S20). All RT-qPCRs were performed on a Qiagen Rotor-Gene Q with the QuantiNova PCR kit using 1 µL of cDNA and 2.5 µM primers per 10 µL reaction. The number of gene copies was averaged across four technical replicates for each sample. Comparisons between control (ds-*eGFP*) versus treatment (*ds-lef-4, -lef-8, -lef-9, -MdBV-2-1, -MdBV-2-2, -MdBV-5,* or *-MdBV-11*) knockdown sample means were evaluated for significance with a two-tailed unpaired *t*-test in RStudio (v2023.06.0 + 421). For samples with confirmed knockdowns, RNA quality was assessed with an Agilent-2100 Bioanalyzer at the University of Georgia Genomics and Bioinformatics Core (GGBC) before paired-end standard directional library construction and RNA sequencing on the Illumina NovaSeq 6000 platform (Novogene).

### RNA-seq and differential expression analysis of RNApol knockdown samples

Unix-based transcript assembly, quantification, and quality assessment was performed on the RNA-seq data from the prepared MdBV RNApol and control knockdown samples using the University of Georgia’s Georgia Advanced Computing Resource Center (GACRC) Sapelo2 cluster. RNA sequence data quality was first assessed with FastQC (v0.11.9) (65), before and after paired-end trimming of the raw sequence reads with Trimmomatic (v0.39) (66), to remove adapter sequence and cull poor quality base calls. RNA-seq reads were aligned to the iyMicdemo2.1a genome assembly with hisat2 (vn3) (67). Assembly of transcripts and expression quantification was performed for each sample by stringtie (v3.0.0) (67). Transcript abundance estimates and table FPKM counts for Ballgown were also created with stringtie (67). In RStudio (v2023.06.0 + 421), Ballgown (v2.38.0) was utilized to identify differentially expressed genes (67). For each gene, FPKM metrics in treatment samples were compared to control samples. Genes with a q-value ≤ 0.05 and a calculated fold change ≥ 2 were reported to exhibit combined significance, while genes with a significant q-value (<0.05) but a fold change < 2 were reported to exhibit a statistically significant difference in expression. Genes with a q-value > 0.05 were reported to exhibit a non-significant difference in expression, while genes with FPKM values below measurable levels were reported as expression not measurable.

### Baculovirus and nudivirus promoter motif searches

Baculovirus early gene motifs (the TATA box TATA[A/T]T[A/T], followed by one of the two early motifs CA[G/T]T or CGTGC within 40 nucleotides downstream) (35), the baculovirus late gene motif ([A/T/G]TAAG) (33–35), a candidate nudivirus early gene motif (ATA[G/C]G[G/C]TAT) (18), and a candidate nudivirus late gene motif (TTATAGTAT) (36) were searched for in sequences 250 bp upstream and 50 bp downstream of transcription start sites of all predicted genes in *M. demolitor*. These sequences were extracted from the genome using the txdbmaker R package (v1.6.2) (68). The presence or absence of promoter motifs was identified using grep and tallied. Binomial or χ² tests conducted in Prism (v10.5.0) compared observed and expected frequencies for these motifs in known MdBV early nudivirus-like genes, late nudivirus-like genes, virulence genes on proviral segments, and all eukaryotic genes.

### Screen for candidate genes from the BV ancestor that are absent in known nudiviruses

Transcripts from the ds-*eGFP*-treated RNA-seq data were aligned to the chromosome-level *M. demolitor* genome assembly with stringtie (67). GffCompare (v0.12.6) was then utilized with -r, -X, and -C options to tag isoforms from the RNA-seq data as overlapping, matching, or novel and combine them with the pre-existing annotation in a gtf file output (67). Using the .combined.gtf output file generated by GffCompare, the RNA-seq data sets for the ds-*eGFP* and RNApol knockdown treatments were also re-analyzed to assess transcription levels of the gene models in the combined gtf file. GffCompare generated a combined.gtf file that was used to identify genes that were differentially expressed between *M. demolitor* ovaries from stage 1 pupae and day 1 adults using previously generated RNA-seq data (19, 67). The chromosome-level genome assembly and GffCompare generated combined.gtf files that were also used to identify genes that were differentially expressed in four different *M. demolitor* tissue samples (day 1 adult ovaries, teratocytes, day 1 adult venom glands, and whole-body larvae [last instars]) that also used previously generated RNA-seq data (14, 15). The combined.gtf gene predictions were translated using Transdecoder to identify gene models for predicted open reading frames greater than 100 amino acids in size (69). These gene models were then evaluated based on the number of introns predicted by GffCompare (cutoff: intron # =0) and their expression in the RNA-seq data sets for the RNApol knockdown experiment (cutoffs: q-value < 0.05, FC < −2), ovaries from stage 1 pupae and day 1 adults (cutoffs: q-value < 0.05, FC > 2 in adult ovaries vs. Stage 1 ovaries), and the four different tissue samples (cutoff: ovary TPM divided by the sum of ovary, teratocyte, venom gland, and whole-body larvae TPMs > 0.99). Genes that met cutoff criteria for each of the above comparisons were manually annotated in JBrowse Apollo (https://i5k.nal.usda.gov/tools/jbrowse-and-apollo).

We expected that any gene from the BV ancestor in *M. demolitor* would not be present in insects outside the Microgastroidea. Thus, predicted protein sequences from JBrowse for each gene that met each of the above cutoff criteria were next assessed with TBLASTN (v2.16.0) on the GACRC Sapelo2 cluster with NCBI nr blast database updated February 3, 2025 to search for matches across all Insecta (taxid 50557) (61). Genes with a percent identity > 50%, a query coverage of > 60, and an e-value of < 2 × 10⁻^6^ in insects outside the Microgastroidea were discarded. To determine if each retained gene had additional homologs in *M. demolitor,* a TBLASTN (v2.16.0) search was run on a nucleotide database created from the chromosome-level genome assembly with makeblastdb (v2.16.0) (61). Genes with no quality hits to other genes in *M. demolitor* or other wasps in the subfamily Microgastrinae were considered single-copy candidate, new nudivirus-like genes that were named “MdBV-” plus a unique number. Genes with hits to already annotated genes in *M. demolitor* or other microgastroid braconid species were considered members of a family if also supported by sequence alignments. If homologs were discovered through TBLASTN, each potential gene family member was manually annotated using Apollo on the USDA’s Jbrowse genome browser for *M. demolitor* to create a predicted gene model (61). The predicted protein sequences from these models were aligned with other putative family members using Muscle (v5.1.0) to confirm gene family identity (62). Some additional gene family members were also discovered when viewing gene expression tracks in JBrowse. Some putative MdBV genes had nearby areas of expression that were of a similar size and showed ovary-specific expression when viewing RNA-seq data in Jbrowse (potentially originating from tandemly duplicated genes). These areas of expression were also manually annotated and evaluated as potential family members with Muscle alignment (62). Any potential family members that did not show conserved amino acid similarity with the putative MdBV genes were discarded, while those that showed conserved sequence similarity were annotated with the “MdBV-” convention, with an additional dash and number added on to differentiate family members. Once added to the final genome annotation, the pupal stage 1 and RNApol knockdown RNA-seq data were re-analyzed following the previously outlined methods to produce updated expression analysis results that enabled classification as early or late genes.

Each of the candidate, new nudivirus-like genes in *M. demolitor* was compared to genes in other related wasp species. Nucleotide blast databases were created using makeblastdb from BLAST+ (v 2.16.0) on Sapelo 2 with the following genomes downloaded from NCBI: *Microplitis mediator* (GCA_029852145.1), *Microplitis manilae* (GCA_030273425.1), *Cotesia congregata* (GCA_903981915.1), *Cotesia glomerata* (GCA_020080835.1), *Cotesia ruficrus* (GCA_048537615.1), *Chelonus insularis* (GCA_013357705.1), *Chelonus formosanus* (GCA_028641665.1), *Diachasmimorpha longicaudata* (GCA_034640455.1), and *Fopius arisanus* (GCA_000806365.1), with *F. arisanus* and *D. longicaudata* serving as outgroups, since these two taxa do not contain BVs (61). Using TBLASTN (v2.16.0) on Sapelo 2, MdBV protein sequences were blasted against each custom database. Blast hits with a percent identity > 50%, a query coverage of > 60, and an e-value of < 2 × 10⁻⁹ were considered orthologs of the MdBV genes.

### Proteomic analysis of MdBV virions for candidate, new nudivirus-like gene products

We reanalyzed previously reported proteomic data sets for MdBV virions (22) to determine if any of the known nudivirus genes that were revised by the new genome assembly or candidate, new nudivirus genes encoded structural proteins. Detailed methods previously described how the MdBV virions samples were prepared, processed, and analyzed (22). In brief, two separately collected and sucrose-gradient-purified samples of MdBV virions were fractionated into envelope and nucleocapsid fractions. Fractions were lyophilized and resuspended in deionized water, and proteins in each sample acetone were precipitated. Protein pellets were then solubilized in SDS-PAGE sample buffer, electrophoresed on 4%–20% Tris-Glycine gels (Bio-Rad), and in-gel digested with trypsin (20 µg/mL) overnight in 20 mM ammonium bicarbonate. Tryptic fragments were extracted using 50% acetonitrile and 0.1% trifluoroacetic acid and vacuum dried. Samples (4 µL) were then analyzed using an Orbitrap Elite mass spectrometer, coupled to an Easy-nLC II Liquid Chromatography (LC) instrument (Thermo Fisher Scientific). Nanospray ionization was performed with a voltage of 2 kV and capillary temperature of 200**°**C. The Orbitrap mass analyzer was used to provide resolutions of 120,000 and 30,000 for MS and MS/MS analyses, respectively. Briefly, a cycle of one full-scan mass spectrum (300–2,000 m/z) was performed, followed by continuous cycles of CID and HCD MS/MS spectra acquisitions of the two or five most abundant peptide ions throughout the LC separation until the candidate ions were exhausted. Data were acquired using Xcalibur software (Thermo Fisher Scientific). MdBV proteins were identified by searching against a custom database using Mascot 2.6 (Matrix Science Inc.). Only genes with >2 unique peptides were considered positively detected. Unique peptide counts for each gene product detected in virions were pooled across replicates for the envelope and nucleocapsid fractions and then compared by a two-tailed binomial test to the expected frequency of 0.5 if not significantly more abundant in one or the other as previously done for the known nudivirus-like genes in the earlier study (22). We classified each of the nudivirus-like gene products as an envelope (E) or nucleocapsid (N) protein if the binomial test was significant (*P* ≤ 0.05) or as a virion protein (E/N) if peptide counts did not significantly differ between fractions.

### Transmission electron microscopy

The impact of the ancestral RNApol knockdown on virion production and the functional roles of putative MdBV genes were further studied by TEM imaging of the mature ovaries as previously described (20). The RNApol was knocked down as previously described. RNAi knockdowns of a select few MdBV genes was completed in the same manner as described for the RNApol genes: *MdBV-5* and *MdBV-11* dsRNA was made for single-gene knockdowns in *M. demolitor*, while *MdBV-2-1* and *MdBV-2-2* dsRNA was used simultaneously for a knockdown of the *MdBV-2* family (Table S19). Parallel injections with ds-*eGFP* were used in all cases as a control. Following dsRNA injections, wasps' ovaries were dissected in PBS within 24 h of emergence as adults, and the ovaries were separated into two 1.5 mL tubes per individual. One ovary was used to verify gene knockdowns with RNA extraction and qPCR with target gene primers as outlined in previous sections. The other ovary from each wasp was fixed in 3% glutaraldehyde in phosphate buffer (pH 7.0) at 4°C. Fixed ovaries with confirmed knockdowns were rinsed with the phosphate buffer and post-fixed with 1% osmium tetroxide. Block staining of fixed ovaries was performed with 0.5% uranyl acetate before dehydration in graded ethanol solutions, 100% acetone, and 100% propylene oxide. Ovaries were embedded in Spurr’s resin before thin sectioning with a microtome. Slices were mounted on copper grids and post-stained with uranyl acetate and lead citrate before imaging with a JEM-1011 TEM at the University of Georgia’s Georgia Electron Microscopy Center.

Calyx cells were characterized by phases of viral production as described by Lorenzi et al.: Phase 1, where envelopes appear *de novo* in the cell nucleus; Phase 2, where electron-dense, barrel-shaped nucleocapsids are first seen near envelopes; Phase 3, where assembled virions are organized at varying levels in parallel arrays; and Phase 4, where the nucleus fills with mature, elongated virions before lysing (20). Within one ovary, there can be variation in stages and exceptions to phenotypes; hence, the most common cell morphologies are what we have reported.

### Novel promoter motif search in late nudivirus-like genes

Regions surrounding the transcription start site (TSS; 250 bp upstream and 50 bp downstream) of all nudivirus-derived genes that were downregulated by RNApol knockdown (q-value < 0.05) were used as input for MEME (v5.5.9) for novel ungapped motif discovery (70). A 20 bp motif named “MdBV late” was used in FIMO (v5.5.9) to scan all TSS regions in the *M. demolitor* genome with a *P-*value cutoff of 0.001 and a background nucleotide frequency model made with fasta-get-markov from the MEME suite with non-default settings of –best-site –norc and a “background” (70). The same criteria were then used to search for TSS regions in the *C. congregata* genome and the genomes of Tipula oleracea nudivirus, Heliothis zea nudivirus 1, Oryctes rhinoceros nudivirus, and Autographa californica multiple nucleopolyhedrosis virus.

## Data Availability

Accession numbers for all data used are described in the text, with raw sequencing data available through the NCBI SRA database. This genome assembly, WGS Project JANIEV01, is represented as BioSample SAMN29911678 with identical records in GenBank as accession GCA_026212275.2 and RefSeq as accession GCF_026212275.2 with the name iyMicDemo2.1a. NCBI’s Genome Data Viewer is accessible at: https://www.ncbi.nlm.nih.gov/gdv/browser/genome/?id=GCF_026212275.2. Data download is available through an FTP site: https://ftp.ncbi.nlm.nih.gov/genomes/all/GCF/026/212/275/GCF_026212275.2_iyMicDemo2.1a/. Curation of the assembly, visualization, manual annotations with Apollo, and consolidated sequence-based resources are hosted by the i5K Workplace (https://i5k.nal.usda.gov/organism/836559). The MdBV RNApol subunit knockdown data are available under BioProject accession number PRJNA1480048.

## SUPPLEMENTAL MATERIAL

Supplemental figures jvi.00506-26-s0001.pdf Fig. S1 to S9.

Supplemental tables jvi.00506-26-s0002.xlsx Tables S1 to S20.

ASM does not own the copyrights to Supplemental Material that may be linked to, or accessed through, an article. The authors have granted ASM a non-exclusive, world-wide license to publish the Supplemental Material files. Please contact the corresponding author directly for reuse.
