# Supplementary material for: A chromosome-scale genome assembly and functional studies implicate the viral RNA polymerase in transcription of most Microplitis demolitor bracovirus late genes
Source: J Virol. 2026 Jul 17;100(8):e00506-26. doi: 10.1128/jvi.00506-26 (PMC13483298; doi:10.1128/jvi.00506-26)
Supplement: Supplementary file 1 [file jvi.00506-26-s0001.pdf]

Supplemental Material

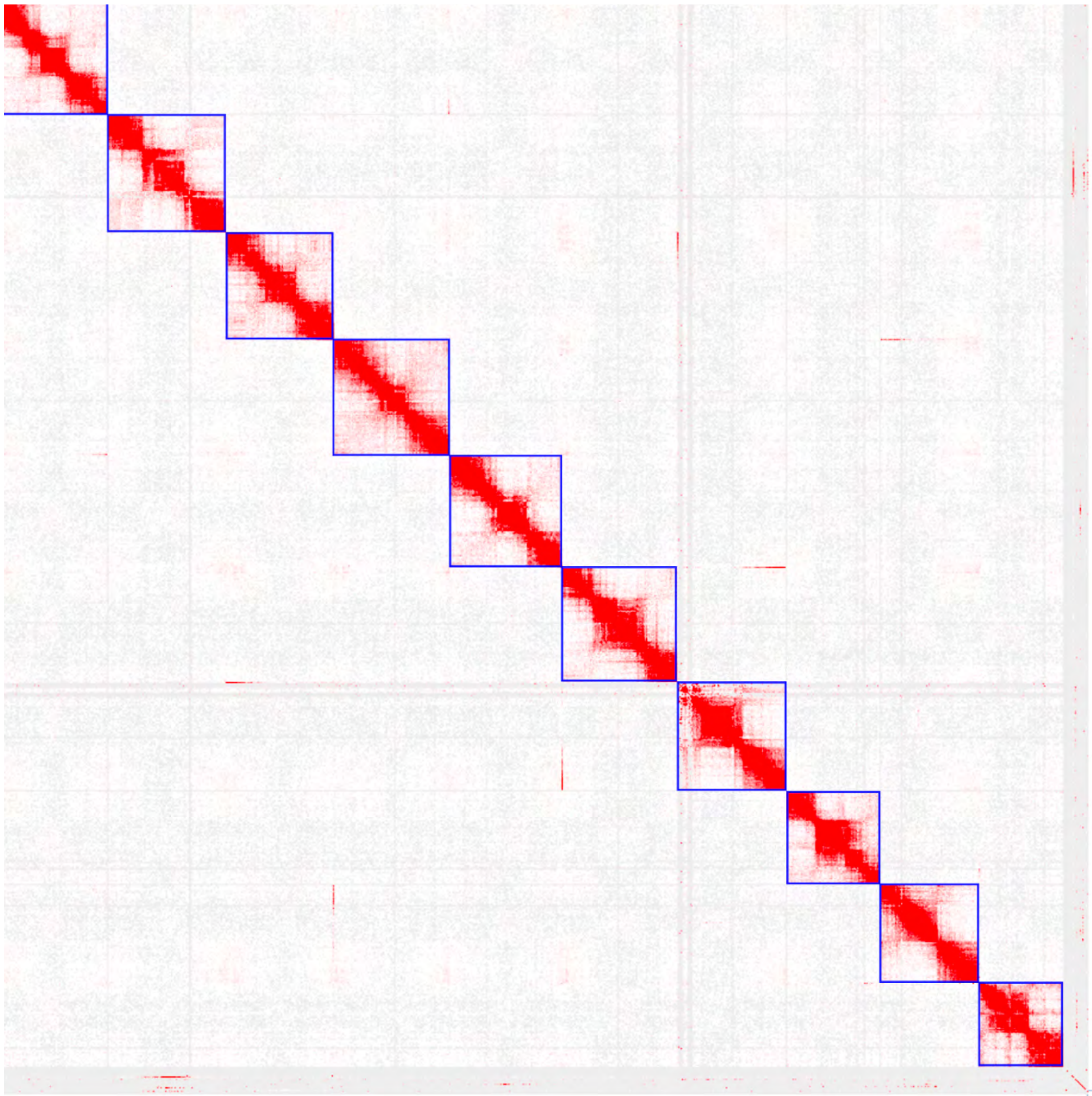

FIG S1 Hi-C contact map for the *M. demolitor* genome. The iyMicDemo2.1a genome assembly yielded 10 chromosomes plus 21 unplaced scaffolds with an overall genome size of 225.9 Mb. Chromosomes ranged in size from 17,637,187 bp to 24,747,562 bp. A BUSCO qualitative assessment of genome completeness found 95.5% of complete single copy, 0.9% duplicated, and 0.4% fragmented genes in the Hymenoptera data set from NCBI containing 5,991 genes with 2.9% of genes missing.

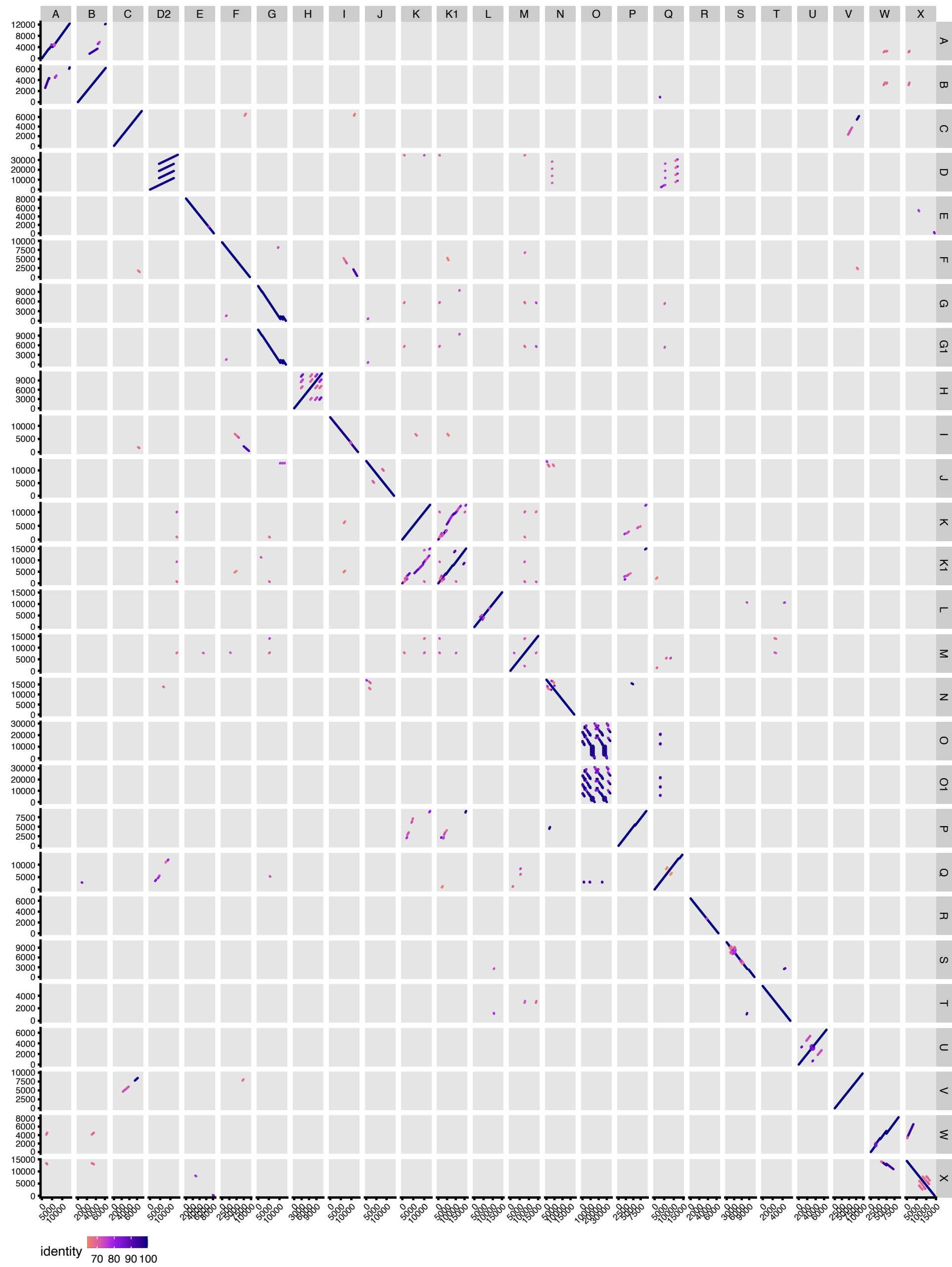

FIG S2 Dot plot comparing MdBV proviral segments (A-X) from the iyMicdemo2.1a and Mdem2 assemblies. Proviral segments from the Mdem2 assembly (scaffold-level) are on the x-axis, while proviral segments from the iyMicdemo2.1a assembly (chromosome-level) are on the y-axis. Dotted lines indicate similar regions identified by BLASTn with default parameters and an e-value cut-off of 1e-20, and color signifying % sequence identity of alignments. Differences in the strand used for query or subject proviral segments results in positive or negative slopes in diagonal lines indicating alignments. Some neighboring segments share regions of similarity, e.g. K and K1, G and G1, O and O1, A and B, and X and W, while Segment O and O1 contain internal repeats. Proviral segment boundaries for the new assembly can be found at https://doi.org/10.15482/USDA.ADC/28590425. Scale is expressed in nucleotides for each segment.

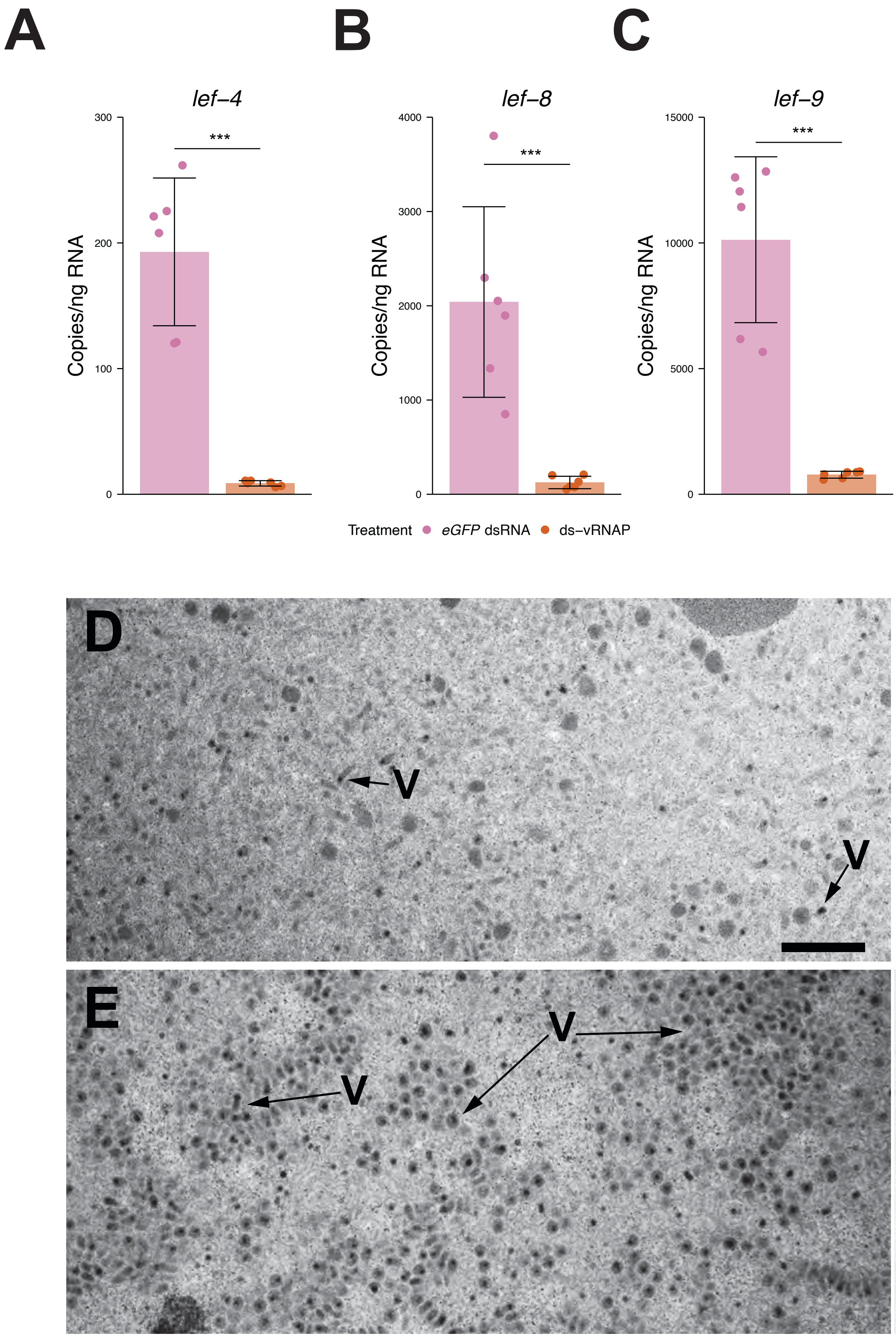

A
B
C
lef–4
lef–8
lef–9
Copies/ng RNA
***
300
200
100
0
4000
3000
2000
1000
15000
10000
5000
Treatment
eGFP dsRNA
ds–vRNAP
D
E
V

FIG S3 RNAi knockdown of *lef-4, lef-8,* and *lef-9* disables MdBV virion formation. (A) Transcript abundance of *lef-4* per ng of total RNA in ovaries from day 1 adult females injected with a cocktail of ds-*lef-4,* ds-*lef-8,* and ds-*lef-9* or ds-*eGFP* (control) as Stage 1 pupae. Bars show mean transcript copy ± standard error, solid circles indicate number of females examined, and asterisks indicate treatment significantly differed (two-tailed t-test, ***, $p<0.001$). (B, C) Transcript abundances of *lef-8* and *lef-9* with treatments, bars, sample sizes and statistical analyses as defined in A (two-tailed t-test, ***, $p<0.001$). (C) TEM image of a calyx cell nucleus from a day 1 adult female after *lef-4, lef-8,* and *lef-9* knockdown. Only small numbers of MdBV virions (V) consisting of an envelope surrounding a nucleocapsid are visible. (D). TEM image of a calyx cell nucleus from a day 1 adult control female treated with ds-*eGFP*. The nucleus contains an abundance of MdBV virions in close proximity to one another. Scale bar in C equals 1 μm with magnification the same in D.

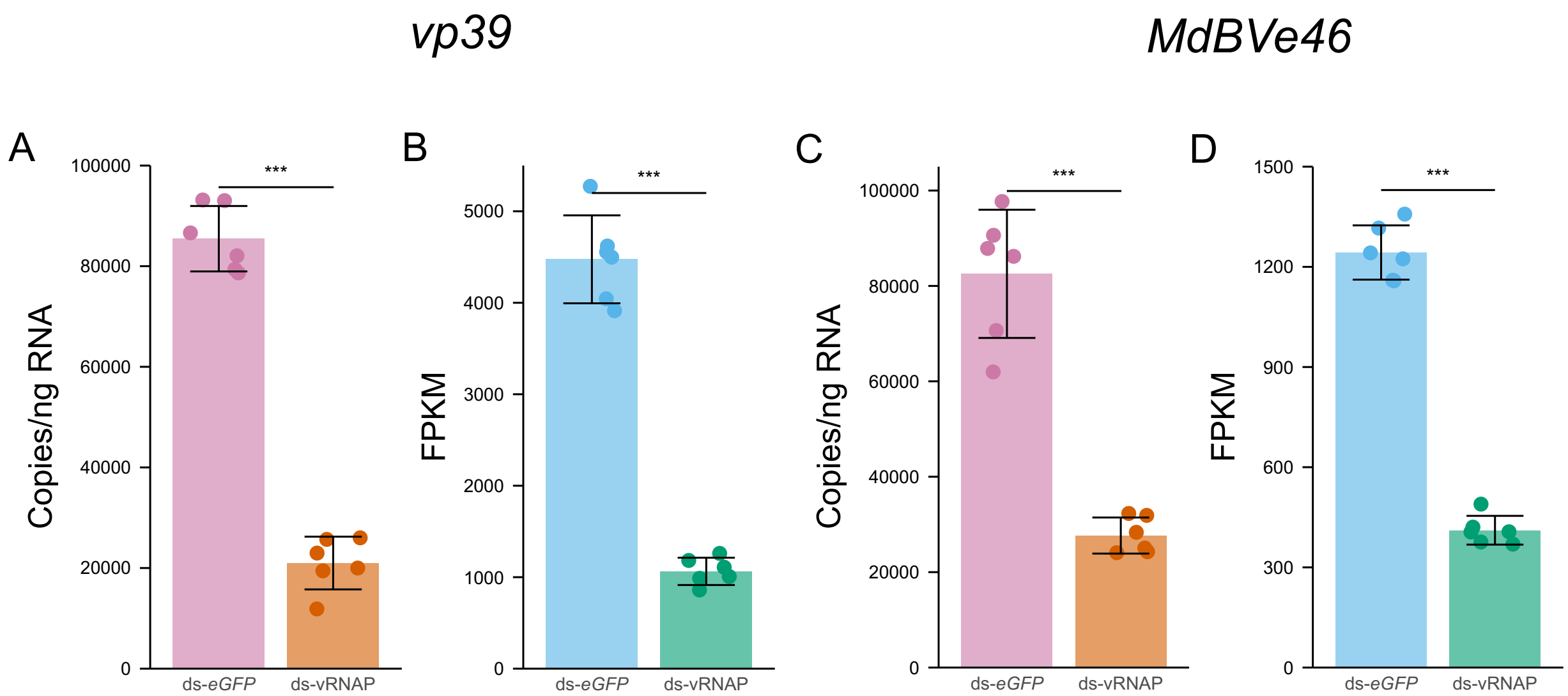

FIG S4 Effect of MdBV RNApol knockdown by RNAi on transcript abundances of *vp39* and *MdBVe46* as measured by RT-qPCR versus FPKM values from RNA-seq data. (A) Transcript abundance of *vp39* per ng of total RNA in ovaries from day 1 adult females injected with a cocktail of ds-*lef-4*, *lef-8* and *lef-9* or ds-*eGFP* (control) as Stage 1 pupae. Bars show mean transcript copy ± standard error, solid circles indicate number of females examined, and asterisks indicate treatment significantly differed (two-tailed t-test, ***, $p<0.001$). (B) Transcript abundance of *vp39* as measured by FPKM (two-tailed t-test, ***, $p<0.001$). (C) Transcript abundance of *MdBVe46* as measured by RT-qPCR. Treatments, bars, sample sizes and statistical analysis as defined in A (two-tailed t-test, ***, $p<0.001$). (D) Transcript abundance of *MdBVe46* as measured by FPKM (two-tailed t-test, ***, $p<0.001$).

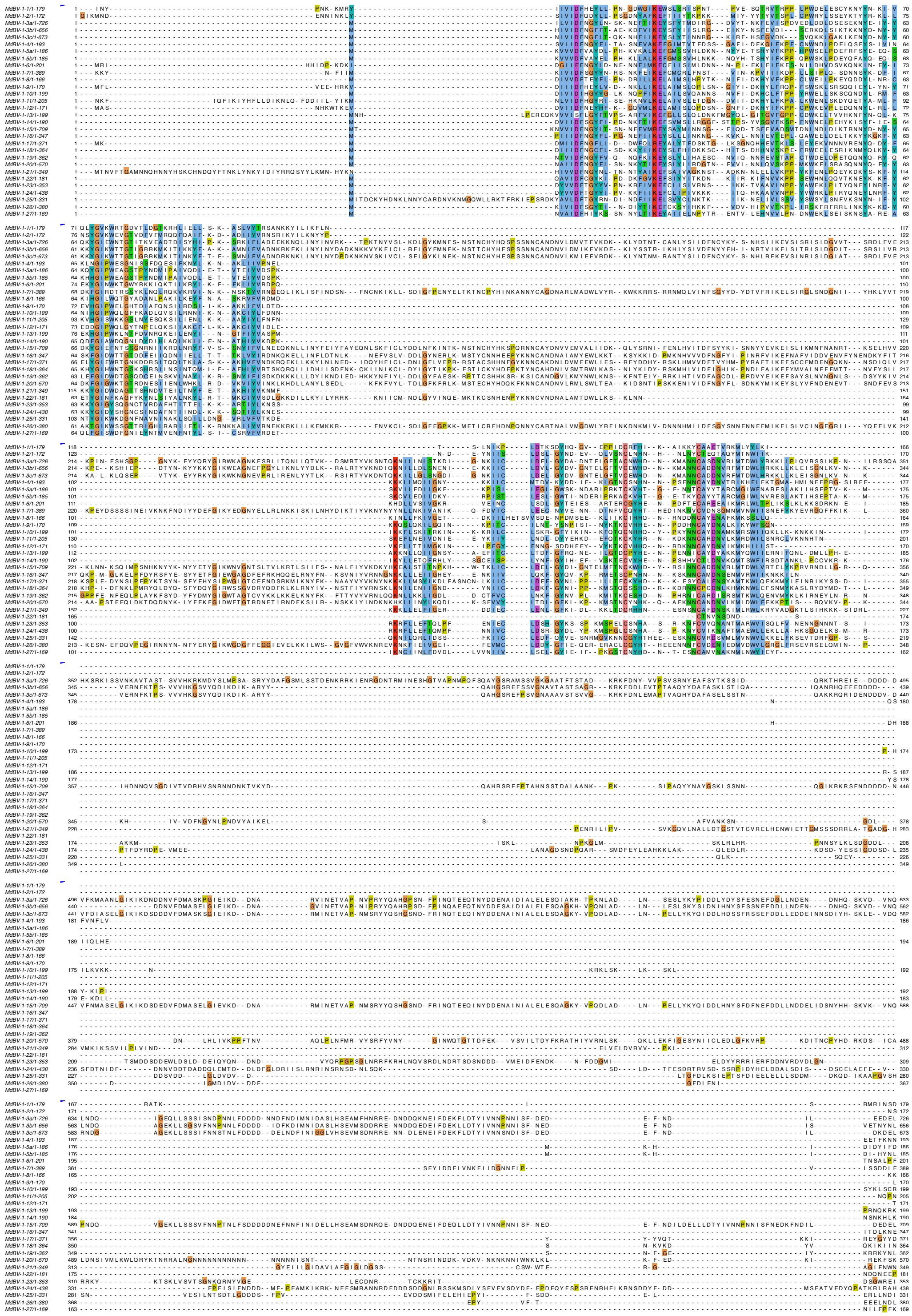

FIG S5 Domain conservation in the MdBV-1 family. Colors in the alignment highlight conserved residues and amino acid residue characteristics. Numbers indicate relative positions of aligned residues in each protein.

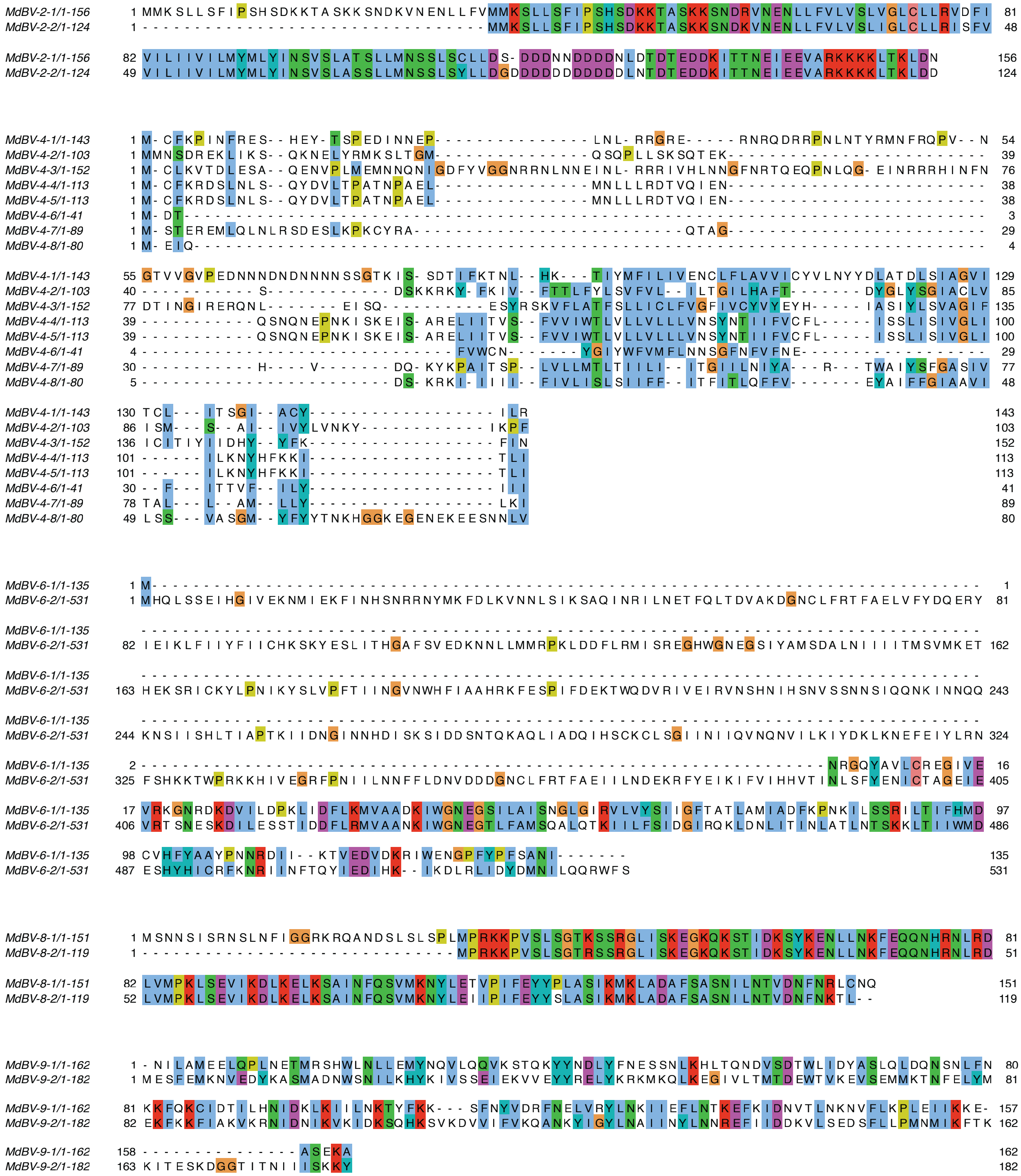
MdBV-2-1/1-156
MdBV-2-2/1-124
MdBV-4-1/1-143
MdBV-4-2/1-103
MdBV-4-3/1-152
MdBV-4-4/1-113
MdBV-4-5/1-113
MdBV-4-6/1-41
MdBV-4-7/1-89
MdBV-4-8/1-80
MdBV-6-1/1-135
MdBV-6-2/1-531
MdBV-8-1/1-151
MdBV-8-2/1-119
MdBV-9-1/1-162
MdBV-9-2/1-182

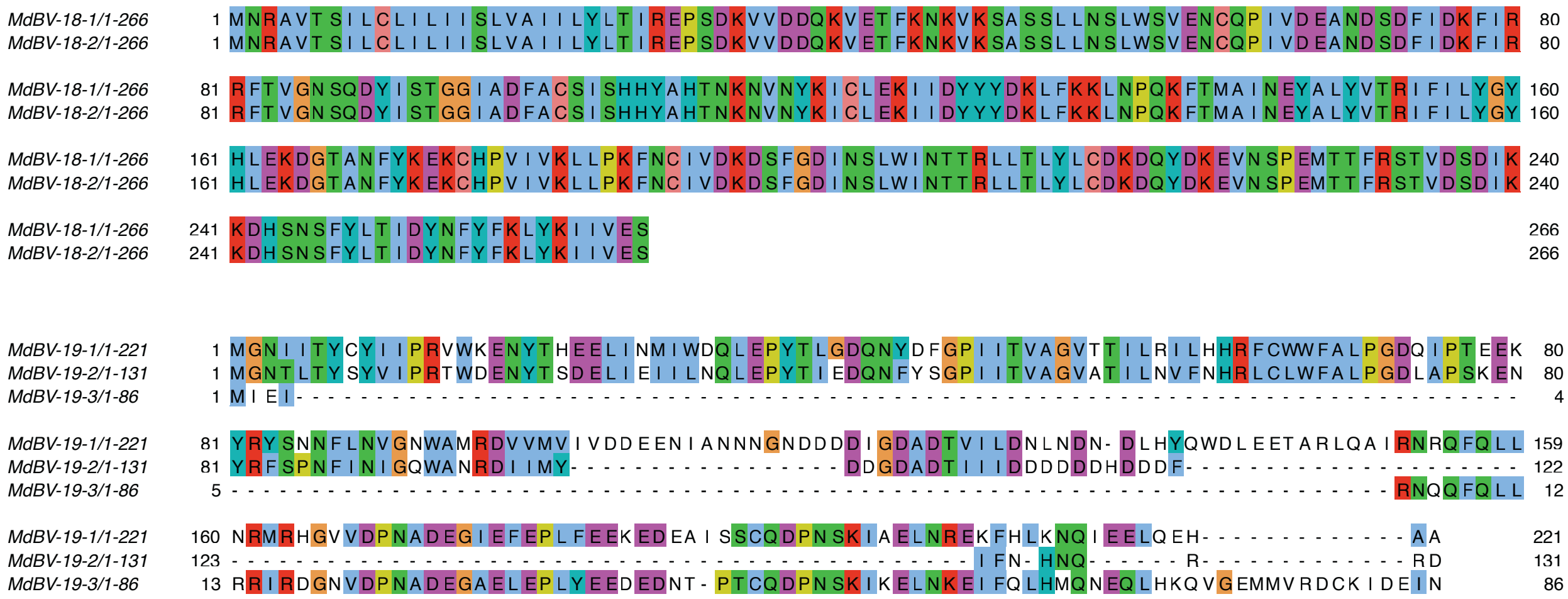

FIG S6 Domain conservation in the *MdBV-2, -4, -6, -8, -9,* and *-19* families. Colors and numbers in the alignment as defined in Fig S5.

```
MdBV-3/1-98     1 KENSFLIMAISKKLFIKELLRNKYLKKYKKEEPEKFEKIFEVINNYEKDSIRNYNDIASCVDKISKILGLSSLEKVIMKKHIKN 84
MdBV-3/1-98    85 KILRLKSSGKKDDK 98

MdBV-5/1-662    1 MKIMITNLADSNIDQSLETFEKFVGKFDLWRNSLSKHLKNDRDILLVFMAKNNPALAITSLFRELNDECKLIYDKVNELSTA 82
MdBV-5/1-662   83 VTNDYLKAYTIGRNIHSLINLHPKLRNTFARYNDMALIYNNYVTKLNDLINESNYAIVYTSDSNDKFMILKDNLIERLNELM 164
MdBV-5/1-662  165 NAGECNEYGHKILMRCKRSLTKFLPCDFSDNFNILPINTFNDDFDSSTNDSSIRTVSTYSSSSSSSTSSSSTSSSSDSSTDE 246
MdBV-5/1-662  247 EALKNIATKMKLGNVNPDNNLSDDSSSDDNLIPDDSSSDDNLVADKNGDKDSSSEDDTTEDSSSDDNLVADKNGDKDSSSED 328
MdBV-5/1-662  329 DTTEDSSSDDNLVADKKGDKDSSSEDDSAENSSSDDNLVADKNGDKDSSSEDDTTEDSSSDDNLVADKNGDKDSSSEDDSGV 410
MdBV-5/1-662  411 NSSSDDNLVADKNGAKDSSSEDDATEDSSSDDNLVADKNGDKDSSSEDDSGVNSSSDDNLTADKNGDKDSSSEDDSSENSSS 492
MdBV-5/1-662  493 DNLVADKNGDKDSSSEDENKINKEKVMLLKAYDSNVDRFSRTLSKIENKTEDADSSDDDSDAQIANNDVNPESSMSDIKDSD 574
MdBV-5/1-662  575 NDEKITEKTETDRNNPTTDNIVKIDDGNAEPSRKRKITQVDGIDNISSSDDGDSEKFDEKEKKMKSSDSSETDSQSSAQVQT 656
MdBV-5/1-662  657 PSNEEN 662

MdBV-7/1-136    1 MYSQSQSQMSDSVNKLKSSLFDIKEFNKLLKQPFESEKSSKNSSNSINNNDKSDNVLIDKELLEELIDCYNNCKHDNTENNST 83
MdBV-7/1-136   84 IISEKIIENNVNVSENKLINSNNLSPNENFNIYDNPALNSLKNLQFNDVAPLT 136

MdBV-10/1-186   1 QIYLYYCNYIIKMFQIDNEKSTTCLKINTYILIVTCIALMSMFAVMYIYNSKASTTDKITIDDVRVISKTVSGLIIASVGVV 82
MdBV-10/1-186  83 GIQAIILVWQQFLLTVVNSDNSTDENKKPGTTSRTKRSLKNSAKRNNEKDNNKIIEKNNSKDEGKSDNILDTIDNVNSVGAM 164
MdBV-10/1-186 165 MEIINSVATTEENNNIEKFIAL 186
```

```
MdBV-22/1-1699     1 YIFINCRIADKTKKLYNMDFLRVMVWINLIVYLTTVIVIIIDICRNSSKAGNNKRKIDVD-QKNEKTTNYPETKIITGAN 79
MdBV-22/1-1699    80 NLNDNKKSIEAAQIDEIPIDSSGKNNSSNMKINEEKSIIEF-GQSNPEKGQTRSRLLEDAANAVAARIKAEKILSKAEMD 158
MdBV-22/1-1699   159 ATEAQTKAQTAVERIKSAISLL-KEGKLNLVENEARAAVGPATDAITAAQAFSRENMPISPVVISEFPNDLSTDSDLVSI 237
MdBV-22/1-1699   238 IAT-VRDNARIVDNVIEKLNSMKGIVNSSQSAAEKVITKVRFILAAADVALEHAKANAVMSEAT-KAQENSEAASAAIDL 315
MdBV-22/1-1699   316 AKFLLKKNKLYFAEKEVQNADRYAANAITAAETFNTDENSITLVY-IDELPTELSKDSDLKSIVSAAREDATEVKNAIIT 394
MdBV-22/1-1699   395 LMSKEAIIKSKQSDAKKAKAAIPFLQ-AAIKAALERHKADRLLPNFKMLASKVEQNSLAATSAIDSAKIALQTDRLDDVE 473
MdBV-22/1-1699   474 TEALKAE-RSAIQAIEEAQNFSYDKNFIRSVVIDELPDELSTDSDMKSIMSKADENAAKVREAIRTIN-NTEKATVMSSQ 551
MdBV-22/1-1699   552 SAAKKAMAAVPLLHVALQAARERKKADQLLPNVKMLASEVEQKSIAASS-AIKSAMTALQNDRLDDVETEALKAESSAIE 630
MdBV-22/1-1699   631 AIQVAQNFSYDQNSISSVDIDDFSNNVSEG-SDLESIKSKVEEDGAEIRKTIETINLKRASIISNQSAAEKAKAAVPLLR 709
MdBV-22/1-1699   710 SAAKVAIERKK-ADAVLFNAIEAEEQSKAASKIIKSAMSLLKDGKLDLVENKALEAINPATAAIEAAQNFSR-DKNSINS 787
MdBV-22/1-1699   788 LDIAELSAILSTDSDLKTIKSKADDDAVNVNAAIGTINTKEASIVSSQSAAEK-AKAAVPLLRSAAKVAIERKKADAVLF 866
MdBV-22/1-1699   867 NAIKAEEQSKAASKIIKSAMSLLKDGKLDLVENK-ALEAINLATAAIEAAQNFSRDKNSINSLDIAELSAILSTDSDLKT 945
MdBV-22/1-1699   946 IKSKADDDAVNVNAA-IGTINTKEASIVSSQSAAEKAKAAVPLLRSAAKVAIERKKADAVLFNAIEAEEQSKAASK-IIK 1023
MdBV-22/1-1699  1024 SAMSLLKDGELDRVENKALEAINPATAAIEAAQNFSRDKNSINSLDIAELSAILSTD-SDLKTIKSKADDDAVNVNAAIG 1102
MdBV-22/1-1699  1103 TINTKEASIVSSQSAAEKAKAAVPLLRTAAKVAIERKK-ADAVLFNAIKAEEQSKAASKIIKSAMSLLKDGKLDLVENKA 1181
MdBV-22/1-1699  1182 LEAINLATAAIEAAQNFSR-DKNSINSLDIAELSAILSTDSDLKTIKSKADDDAVNVNAAIGTINTKEASIVSSQSAAEK 1260
MdBV-22/1-1699  1261 -AKAAVPLLRSAAKVAIERKKADAVLFNAIEAEEQSKAASKIIKSAMSLLKDGELDLVENK-ALEAINPATAAIEAAQNF 1338
MdBV-22/1-1699  1339 SRDKNSINSLDIAELSAILSTDSDLKTIKSKADDDAVNVNAA-IGTINTKEASIVSSQSAAEKAKAAVPLLHVALQAARE 1417
MdBV-22/1-1699  1418 RKNADKRLTNVEMLASEVEQKSI-AASSAIKSAMTALQNDRLDDVETEANNAEHSATEAITAAQKLSIDTHSVRPINIDD 1496
MdBV-22/1-1699  1497 FSAN-VADGSALASIKTKVEDDAAEVMKAIETINLKETSVISHKATAEKAKAAVPFLRIALQVAR-QRKNATERLVKAQK 1574
MdBV-22/1-1699  1575 SAAESAENAKNAAANFSRVEKSEDPSLAESISLQADVALSFALQAI-AYAQIFLSTKRLVDYAATQARKLAKQISDDYTD 1653
MdBV-22/1-1699  1654 IKAIANKLVNESEEASNALAKLEESVNTSMHSARSTQTEASKLVEI 1699

MdBV-23/1-342      1 MLSMSSFYYLIFIITIIFAIIVSFPMTFFRRYLNNKNYVLPTTGPINLEYNSNNVASTEE-FLDLQEPNYVPTPTNDNWESL 81
MdBV-23/1-342     82 QNDQVLSAGTFKSKEPVSEESLNKQLINLNSIDTDTTAD-DKFNDNFKNIVIQTIKQNIKYLLSDNNNYETHLSYPDNYVQD 162
MdBV-23/1-342    163 ILKNMKGNETSGLKDLCN-FGIKILDMHLTSQRKKKFYMNSLINIIKNINKIIVPQVNINDNIPETDNSNWDNTIYRLL-IL 242
MdBV-23/1-342    243 LAMYEYIGYNNLNKLSTCHNVIKSIIQKFHESVGMPFDNDIKFICLTIPTLLTDYLYD-RHNFHDKIKEAVGYLENIFYKVD 323
MdBV-23/1-342    324 YQNNFYFSIYTIFSNELNN 342

MdBV-24/1-96       1 MNSDRPSKRNSLEKYRLIANYNNIQVLTINVSAKYYTRICPDLCCWRFNNYLRVQRNYSTLNLLFDFNTIYNASIPPVMHSNIQ 84
MdBV-24/1-96      85 FTKKFRKMSSVG 96
```

FIG S7 Sequences of the single copy genes *MdBV-3, -7, -10, -18, -22, -23,* and *-24*. Colors and numbers as defined in Fig S5.

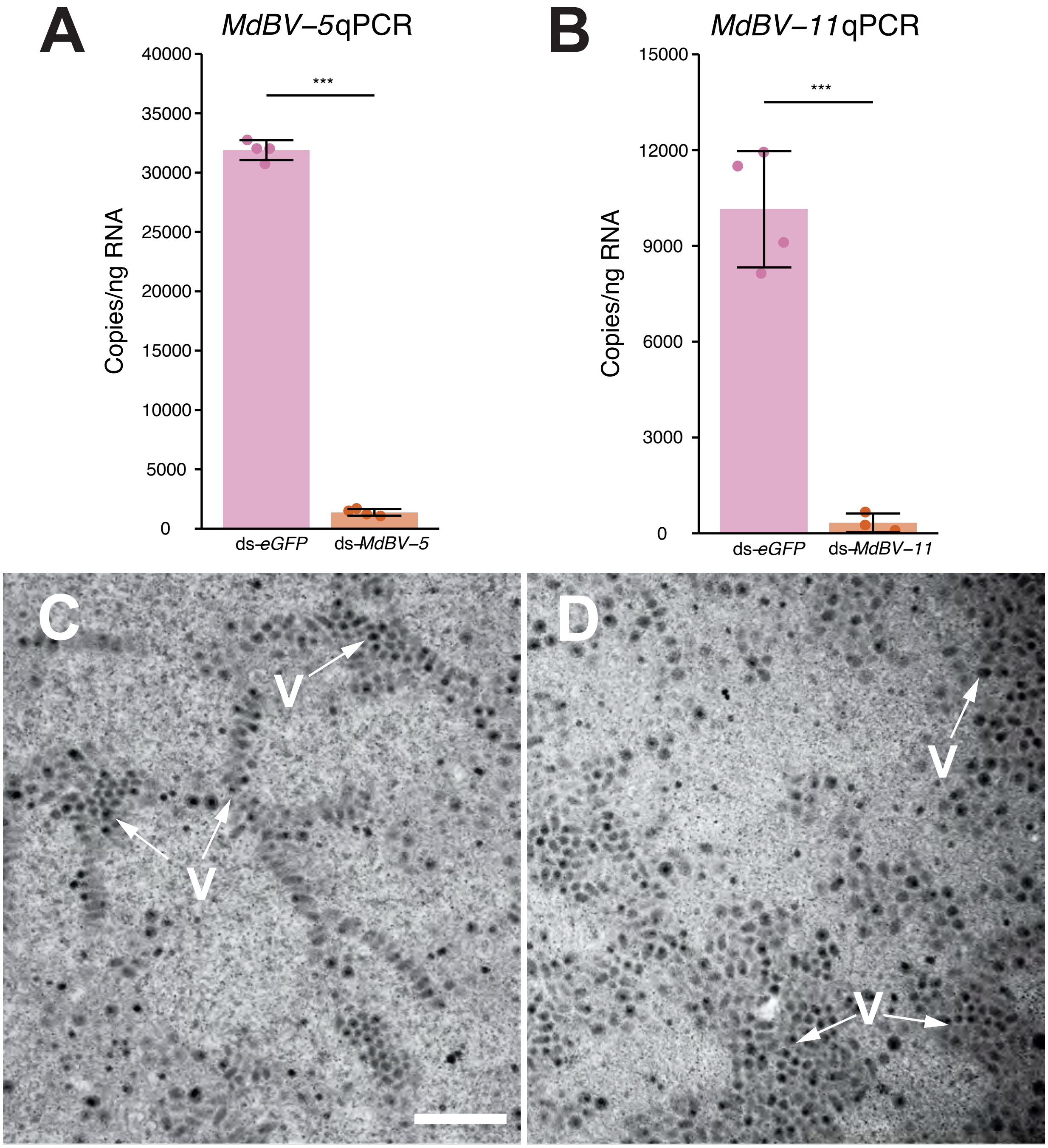

FIG S8 RNAi knockdown of MdBV-5 and MdBV-11 has no visible effect on MdBV virion formation. (A) Transcript abundance of MdBV-5 per ng of total RNA in ovaries from day 1 adult females injected with a cocktail of ds-MdBV-2-1 and ds-MdBV-2-2 or ds-eGFP (control) as Stage 1 pupae. Bars show mean transcript copy ± standard error, solid circles indicate number of females examined, and asterisks indicate treatment significantly differed (two-tailed t-test, **, P<0.01). (B) Transcript abundance of MdBV-11 with treatments, bars, sample sizes and statistical analysis as defined in A (two-tailed t-test, ***, P<0.001). (C, D) TEM images of calyx cell nuclei from day 1 adult females after MdBV-5 and MdBV-11 knockdown. Both treatments show an abundance of MdBV virions that show no morphological alterations. Scale bar in C equals 1 μm with magnification the same in D.

Name	Starting coordinate	*p*-value	Sites		
iyMicDemo21aOGSv2.0-000254	274	1.23e-8	TTTAACGTAC	GCTTTATATTAGATGGGTAA	TATTATA
iyMicDemo21aOGSv2.0-000035	198	2.44e-8	TTTTATAGTT	GCGTTATAGTAGAAGACAAA	AATTTATTAA
iyMicDemo21aOGSv2.0-000223	232	5.02e-8	AAAAAAGTTA	ACCTTTTATTAGACGAAAAA	GCGTAAAATT
iyMicDemo21aOGSv2.0-000236	228	7.76e-8	AAAATTCTTT	GCTATTTATTAGAAGAAAGA	AAAATATATT
iyMicDemo21aOGSv2.0-000233	98	1.41e-7	TAAAATTTCC	ACTTTATATTAGTTGAGAAA	AACTCCATAA
iyMicDemo21aOGSv2.0-000202	254	1.73e-7	TTTTTATTTT	GCTTTATTATAGAAGACTAA	AACGATAAAT
iyMicDemo21aOGSv2.0-000034	233	2.09e-7	TTTTTTTTAC	ACTTTATTATAGAAGACAAA	ACAAACATAT
iyMicDemo21aOGSv2.0-000030	250	3.72e-7	TCAATAACTG	AACTTATATTAGAAGAGTAA	AACAGATTCA
iyMicDemo21aOGSv2.0-000198	257	4.49e-7	TTTTTTATTC	GTCTTATTGTAGAAGACAAC	GAGTTGTGCG
iyMicDemo21aOGSv2.0-000237	202	5.34e-7	ATAAATTATA	TGATTATATTAGAAGCGAAA	CAGAATAGCA
iyMicDemo21aOGSv2.0-000053	229	5.34e-7	TTTTTTTAAT	GCTTTTATTTAGACGACACA	AATACATATT
iyMicDemo21aOGSv2.0-000199	248	5.34e-7	TTTTTTATTC	GTCTTATTATAGAAGACAAC	GAGTTGTGCG
iyMicDemo21aOGSv2.0-000259	220	6.38e-7	AATTATTCAT	TACTTATATTAGAAGAAAGA	ATTCTAATAA
iyMicDemo21aOGSv2.0-000028	220	9.00e-7	AAAAAAGTAA	CGTTTATTTTAGAAGAGTAG	AAATAATATA
iyMicDemo21aOGSv2.0-000201	233	1.06e-6	AATATTTCAT	CTGTTATATTAGAAGATAAA	GAAAATTCAT
iyMicDemo21aOGSv2.0-000200	257	1.06e-6	TTTTTTATTT	TCTTTATTATAGAAGCCAAC	GAGTTATGAA
iyMicDemo21aOGSv2.0-000246	207	1.26e-6	TTTAAACTAT	ACTTTTTATTAGAAAGCCCA	AAAATTTGTA
iyMicDemo21aOGSv2.0-000208	239	1.26e-6	TATTTTACTA	TCGTTATATTAGAAGAAATA	TAACTTTATA
iyMicDemo21aOGSv2.0-000242	213	1.48e-6	TAAAGTCAAC	TCTTTCTTATAGAAGGGCAA	GAAAGTCGAA
iyMicDemo21aOGSv2.0-000065	277	2.03e-6	AAAAAAATAA	CGTTTATATTAGAAGACTTA	AAAT
iyMicDemo21aOGSv2.0-000227	226	4.32e-6	TTTTTAGTGT	GCTATAAATTAGTAGAAGAA	TTTTTTATAT
iyMicDemo21aOGSv2.0-000056	260	4.99e-6	ATAATTTTTC	GCTTCTTATTAGACAAGAGA	TATCTCAGAT
iyMicDemo21aOGSv2.0-000219	273	5.75e-6	TTAATTAATA	TATTTATATTAGACGTGAAA	AAAAAATT
iyMicDemo21aOGSv2.0-000247	226	7.57e-6	TAATTAAAAA	GTTTTATATTAGTATAAAAA	AATAATTTTG
iyMicDemo21aOGSv2.0-000084	229	9.90e-6	TTAAATAATA	ACTATATTATAGAGGACAAA	CAATTTAGTT
iyMicDemo21aOGSv2.0-000257	227	9.90e-6	GTTTTAGAGA	GTATTATATTAGTACCGAAA	GAAATACTAT
iyMicDemo21aOGSv2.0-000052	248	9.90e-6	TTTCTTGAAT	GCCTTTCCTTAGATGAGAGG	ATATTCAAAT
iyMicDemo21aOGSv2.0-000283	252	1.87e-5	AAAAATAATC	GTTTTATAATAGAGTAGAAA	CATAACTATT
iyMicDemo21aOGSv2.0-000033	227	1.87e-5	AAAAAATTCG	AGCTTATATTAGAGAAGCAT	AAAAATGGCG
iyMicDemo21aOGSv2.0-000277	221	2.11e-5	TTTTTAATTT	ACTATTTATTAGATAAAAGA	CTATTTTTGA
iyMicDemo21aOGSv2.0-000250	228	2.11e-5	AAAAAATGTT	GATATATATTAGTAAAGGAA	GATAAATAAA
iyMicDemo21aOGSv2.0-000051	181	2.11e-5	TTTTTTTAAT	ACCTTTATTTAGATGGTAAG	TATTACATAA
iyMicDemo21aOGSv2.0-000026	235	2.11e-5	TTTTTTTAAT	GTTATTTATTAGACAAAGAA	TATTATTGTT
iyMicDemo21aOGSv2.0-000243	272	2.38e-5	AAAAAGTTGT	TCGTTTACTTAGAAGGTAAA	CGTCTTGAC
iyMicDemo21aOGSv2.0-000037	241	2.38e-5	AAATTTTATT	ACTTTATTGTAGTAAAGTAA	ATTTTGTAAT
iyMicDemo21aOGSv2.0-000289	240	2.68e-5	AAATTTTCTT	CTTATTTATTAGAAAATAAA	AATGGGTTTA
iyMicDemo21aOGSv2.0-000086	241	2.68e-5	AAATTTTCTT	CTTATTTATTAGAAAATAAA	AATGGGTTCA
iyMicDemo21aOGSv2.0-000006	235	2.68e-5	ATAAATTGCA	GAATTCTATTAGAAGAATCA	ATTAGTAATA
iyMicDemo21aOGSv2.0-000091	221	2.68e-5	ATAAATTGCA	GAATTCTATTAGAAGAATCA	ATTAGTAATA
iyMicDemo21aOGSv2.0-000235	230	3.02e-5	TAGGGTTAAT	TTTTTAAAGTAGAAGGAAGC	ACCGTGCAGT
iyMicDemo21aOGSv2.0-000234	228	3.02e-5	TAGGGTTAAT	TTTTTAAAGTAGAAGGAAGC	ACCGTGCAGT
iyMicDemo21aOGSv2.0-000015	146	3.02e-5	AATTTTAAAT	CTATTATATTAGTAGATAAC	GATTTAAATT
iyMicDemo21aOGSv2.0-000161	236	3.02e-5	AAAATTTATT	TCCTTTTCTTAGTACACCGA	TTATAAAAAC
iyMicDemo21aOGSv2.0-000160	180	3.02e-5	ATGTTGTATT	GTGTTATTATAGAAGATAAT	TATTTTTAAT
iyMicDemo21aOGSv2.0-000163	249	3.38e-5	CTTAAACAAT	CTCTTTTATTAGATGTAAAT	AACAATCAAT
iyMicDemo21aOGSv2.0-000046	248	4.23e-5	TTATTTATAT	ACTTATTATTAGATAAAAAA	ATAAAATTAT
iyMicDemo21aOGSv2.0-000180	233	4.23e-5	ATTAGTTTTT	ATTATCTATTAGATGGGATA	ATCGTAGGTA
iyMicDemo21aOGSv2.0-000174	181	4.23e-5	TTATCATACT	GTTATTATTTAGAAAGAAAA	AAAATTTATC
iyMicDemo21aOGSv2.0-000173	234	4.23e-5	ATTTTATTTC	ATGTTTTATTAGAACAATGA	GTTTTTATTA
iyMicDemo21aOGSv2.0-000166	226	4.23e-5	AACTAATTCG	TCTTTATATTAGTACTAGAA	TTTTTGTTAC
iyMicDemo21aOGSv2.0-000245	237	4.72e-5	AAAAAATTAT	ACTTTTTATTAGGAAGCCCA	AATATTTTAA
iyMicDemo21aOGSv2.0-000204	257	5.84e-5	AATGGAAATT	TCTTTCATTTAGAATACAAG	GCTTCGACAA
iyMicDemo21aOGSv2.0-000171	231	5.84e-5	TTAAATAAAT	GTTTTTTATTAGTACTAGAA	TATTGAAAAA
iyMicDemo21aOGSv2.0-000286	239	6.49e-5	AAAAATTATT	ACATTCTATTAGTAACGAAT	ACATTCAAAA
iyMicDemo21aOGSv2.0-000251	237	6.49e-5	CTTTTTTTTC	TGCTTTCATTAGAACGGCAT	CAAAATGAAT
iyMicDemo21aOGSv2.0-000207	226	6.49e-5	TATTATTATT	GTTATTATTTAGATAACAAA	AATATACAAA
iyMicDemo21aOGSv2.0-000032	228	7.96e-5	TTAAAAGTAA	CGGTTATATTAGAGGGACTG	ATGTAAATTA
iyMicDemo21aOGSv2.0-000093	160	7.96e-5	TTAAAATTAT	GTTTTATATTAGCATCCAAC	TTAGGGAGTA

Name	Starting coordinate	*p*-value	Sites		
iyMicDemo21aOGSv2.0-000244	211	8.80e-5	AATAAATTAT	TGCTTTATATAGAAAAGAAG	TTTTCTCTTT
iyMicDemo21aOGSv2.0-000181	223	8.80e-5	AAATTACTGC	AACGTTTATTAGAAGATAGA	ATAATAGCCA
iyMicDemo21aOGSv2.0-000288	95	9.71e-5	GGTCACTAAG	AATTTTTTTTAGATAAAACA	GGATAGCTGT
iyMicDemo21aOGSv2.0-000027	222	9.71e-5	TTTTTTTTTA	CCTATATATTAGACAGAGCT	CATCTGAAAT
iyMicDemo21aOGSv2.0-000083	247	1.07e-4	TTTTTTTTAC	TATATATATTAGATACAAAA	AATGTTTTCT
iyMicDemo21aOGSv2.0-000262	232	1.07e-4	TAATATTTAT	AATATATATTAGACAAAGAA	ATTCACAAAA
iyMicDemo21aOGSv2.0-000011	235	1.07e-4	TGATAAAAAT	TTCTTATTATAGAGGATAAT	TGTTGGTAGA
iyMicDemo21aOGSv2.0-000206	230	1.29e-4	TGCAAAAGTA	TATTTATATTAGATGTATCA	TAAATATACT
iyMicDemo21aOGSv2.0-000087	224	1.55e-4	ATTTTTTTTA	TCTTTATTATAGTAAAGTTA	TTTCTCACTT
iyMicDemo21aOGSv2.0-000280	247	1.55e-4	ATATCTATAT	TCTTTATTTTAGACTCTTAC	AATTGAGTGA
iyMicDemo21aOGSv2.0-000167	235	1.86e-4	TTATTTTAAT	AGCTTTCTTTAGAAGCAATT	GTGATAAATA
iyMicDemo21aOGSv2.0-000287	244	2.03e-4	GTTTTTGTTT	TGTATTTATTAGTATCGAAT	TCGATTTTGA
iyMicDemo21aOGSv2.0-000061	250	2.03e-4	TCAATCAATA	GTTCTATAATAGAATAGAAC	TATTAGAAAA
iyMicDemo21aOGSv2.0-000029	246	2.03e-4	AAAGAACATA	ATCTCATATTAGAATACTAC	AAAAAAAAAA
iyMicDemo21aOGSv2.0-000183	50	2.03e-4	TCTATCCGAA	AACTTATTTTAGTAACTTAA	TAAATATATG
iyMicDemo21aOGSv2.0-000031	167	2.21e-4	GACTATGCAG	ACATACTATTAGTAGGAAAT	AAAATAACAA
iyMicDemo21aOGSv2.0-000050	65	2.83e-4	TTTTTTTTTA	ATCTTTATTTAGATAATAAG	AACAAAATCG
iyMicDemo21aOGSv2.0-000203	211	2.83e-4	TTTATGATAC	TGTTAATTTTAGTCGACTAT	TCTAATTGTA
iyMicDemo21aOGSv2.0-000182	206	2.83e-4	AAATCTAATT	ACCTTAATTTACATGACTAA	TAATTATAAT
iyMicDemo21aOGSv2.0-000229	226	3.07e-4	TTTCGATGTT	TTCTTTTATTAGTAAGACTT	GTCTAGGAAT
iyMicDemo21aOGSv2.0-000178	224	3.07e-4	AATTAAATTA	TATTATAAATAGAAGCGCAA	ATATTATATG
iyMicDemo21aOGSv2.0-000012	252	3.07e-4	ATAAAATTAT	CCCTTTATATAGATGTATGA	ATATTTTAAA
iyMicDemo21aOGSv2.0-000014	220	3.33e-4	ATATTTATAA	TTTATTTATTAGTAATGAAT	GAAATGATAA
iyMicDemo21aOGSv2.0-000168	11	3.88e-4	AAAAATAGAT	GTATAATTTTAGTTGAATAA	AAAATTTAAA
iyMicDemo21aOGSv2.0-000080	218	4.51e-4	TTTTTTTAAC	ACTTATATTTAGATAACGCA	ACGATAAAAT
iyMicDemo21aOGSv2.0-000177	229	4.51e-4	TTAAATATAA	TTTATTAATTAGATCCTAAA	AAAAAAATTT
iyMicDemo21aOGSv2.0-000016	89	5.60e-4	ACTAATTAAT	CGATTAAATTAGAAAAAATT	TTTTATTTCT

FIG S9 MdBV late motif in the 85 nudivirus-like late genes that were down-regulated by MdBV RNApol. The ID for each gene is indicated to the left, while the starting coordinate, p-value and sequence of the 20 bp MdBV late motif follow.
